# Optimization of electrocoagulation removal of a mixture of three azo dyes: spectrophotometric colour characteristics for best operating conditions†

**DOI:** 10.1039/d4ra08485c

**Published:** 2025-02-27

**Authors:** Aditi Sugha, Manpreet Singh Bhatti

**Affiliations:** a Department of Botanical & Environmental Sciences, Guru Nanak Dev University Amritsar Punjab India asugha12@gmail.com mbhatti.dobes@gndu.ac.in

## Abstract

In the present study, a mixture of three azo dyes *viz.* methyl orange, congo red and acid blue-113, was treated using electrocoagulation. Experiments were carried out to optimize the process parameters, namely, pH (5–9), current density (40–100 A m^−2^), treatment time (10–30 min) and electrode type (aluminium, iron or stainless steel) using flexible design structure by combining mixture components and process factors. Under the optimum conditions, a maximum colour removal of 99% and a COD removal efficiency of 81.9% were obtained for the mixture of dyes (33.3 mg L^−1^ each) using stainless-steel electrodes with a specific energy consumption of 87 kW h kg^−1^ COD. ANOVA model statistics showed that the interactions of electrode type with all the dyes were highly significant. The current density demonstrated a significant effect on methyl orange and Congo red removal, whereas treatment time was only significant for removal of Congo red dye. The pH was not found to be a significant process factor for the removal of the mixture of dyes and may be kept within the range of pH 5–9. Colour characterization using spectrophotometric methods at different wavelengths for estimating hue, tinge and luminance is required to check the effectiveness of electrocoagulation treatment of dye mixtures. A thorough literature review of twenty different studies to propose the optimal conditions is the strength of this study. Multi-dimensional convex hull plots have been prepared to show the effect of current density on colour removal efficiency, in which the size of the circle represents current density, the colour of the circle represents electrode type, and each circle is labelled with the corresponding reference on a single graph, which provides an efficient way to visualize the best operating conditions. However, the efficiency of the electrodes to remove colour and COD is largely dependent on the nature of the dye and the concentration of each dye in the mixture.

## Abstract 2

In the present study, a mixture of three azo dyes *viz.* methyl orange, congo red and acid blue-113, was treated using electrocoagulation.

## Graphical abstract

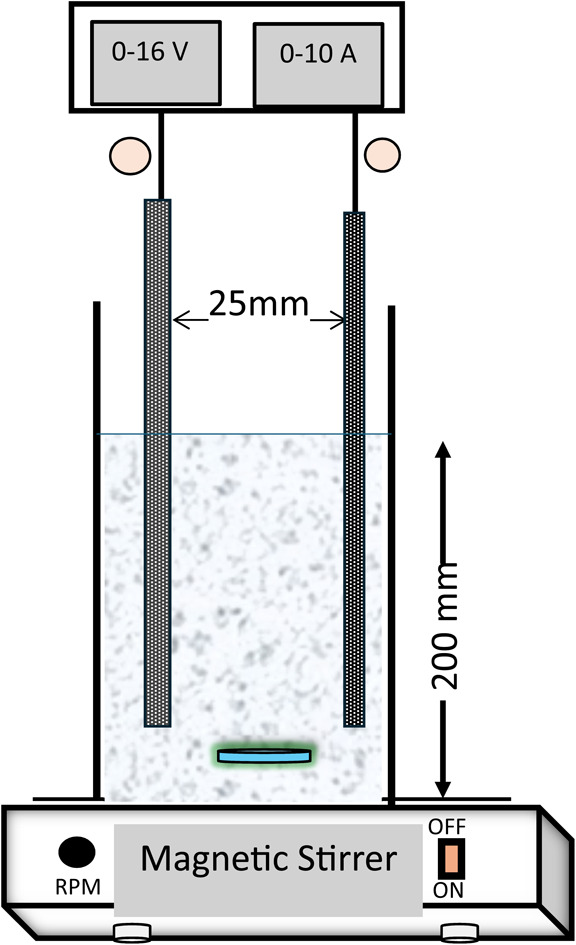

## 1. Introduction

Due to the rapid expansion of industrial activities, the use of colouring agents like dyes is increasing. Dyes are substances that absorb visible light in the wavelength range of 400–700 nm. The chromophore group, which is a delocalized electron system with conjugated double bonds, is the primary structural component that contributes to the light absorption in dye molecules. Dyes are divided into various categories based on their molecular structure and applicability in textile industries. Based on their molecular structure and the presence of chromophores, they are classified as nitro, nitroso, azo, triphenyl methyl, phthalein, indigo and anthraquinone dyes.^1^ Out of all the dyes, azo dyes, which consist of azo links surrounded by substituted aromatic or heterocyclic moieties, are a prominent class of colourants used globally.^2^ These dyes have azo groups (–N=N–) connected to aromatic rings with the help of mono, di, or poly azo groups.^3^ Dyes are toxic to the environment due to their colour and hazardous carcinogenic degradation products like naphthalene, benzamine and other aromatic compounds. To reduce the harmful impact of dyes, it is necessary to treat dye-contaminated wastewater before release into natural water. The electrocoagulation method has become increasingly prominent in recent years owing to its numerous desirable attributes, including simplicity, reliability, and cost-effectiveness, particularly in terms of energy unit pricing.^4^ Electrocoagulation is being widely utilized as a successful method for the removal of a variety of dyes, such as methyl orange,^5^ reactive orange-16,^6^ tartrazine,^7^ direct violet-35 ^8^ and real textile wastewater.^9^ It involves the *in situ* formation of coagulants by the dissolution of metal electrodes like aluminium, iron, and stainless steel. These electrodes result in the production of metal hydroxides at the anode surface and hydrogen gas at the cathode surface. This process combines electrochemistry, flotation, and coagulation techniques to facilitate the electrochemical reduction of water, thereby producing hydrogen gas at the cathode.^7^

The effectiveness of the electrocoagulation process can be enhanced by optimizing the operational parameters, with the design of experiment (DOE) methodology being the most suitable approach for achieving this goal. DOE involves the development of empirical models based upon setting process conditions of independent process variables. By employing the DOE strategy, it is possible to optimize the process in minimum experimental runs. Response surface methodology (RSM) is one of the mathematical and statistical approaches used in model building and process optimization. RSM has emerged as an effective tool for optimizing process parameters in electrocoagulation for the treatment of various types of dyes, such as CI disperse red-343 and Isolan bordeaux-2S-B,^10^ reactive blue-4 ^11^ and textile wastewater.^12^ There are two types of experimental designs: (i) standard designs and (ii) custom designs. Standard designs include one-factor-at-a-time (OFAT) design, factorial design, RSM and mixture design, whereas custom designs include optimal (combined) designs. Optimal design is a framework that is adaptable to support custom models, categoric factors and irregular (constrained) areas. These optimal designs have been effectively applied to optimize the electrocoagulation removal of textile dye wastewater,^13^ surface water,^14^ textile dye bath,^15^ nitroaniline^16^ and cattle slaughterhouse wastewater.^17^

The main objective of the present study is energy-efficient removal of a mixture of three dyes using electrocoagulation treatment. Optimal response surface design was used to optimize the independent process parameters *viz*. pH, current density, treatment time and electrode type along with a mixture of three azo dyes. The absorption spectra and colour characteristics of the dyes before and after treatment were studied to find the best electrode pair. The study is interesting in terms of proposing the best operating conditions for about fifteen other dye studies using convex hull multi-dimensional plots.

## 2. Materials and methods

### 2.1 Chemicals

All the chemicals used in this study were of analytical grade. Methyl orange (MO), congo red (CR), and acid blue-113 (AB113) were procured from Central Drug House (P) Ltd, India. Dye properties and chemical structures along with standard plots are given in Table 1, Fig. S1 and S2,† respectively. A stock solution of 1000 mg L^−1^ of dye was prepared in 1 L distilled water and was subsequently diluted as per working requirements. The solution pH was adjusted using dilute H_2_SO_4_ or NaOH. NaCl was used as an electrolyte to maintain the desired conductivity.

**Table 1:** Properties of dyes

Dye	Molecular weight (g mol^−1^)	Molecular formula	*λ*_max_ (nm)
Acid blue -113	682	C_32_H_21_N_5_Na_2_O_6_S_2_	564
Congo red	697	C_32_H_22_N_6_Na_2_O_6_S_2_	496
Methyl orange	327	C_14_H_14_N_3_NaO_3_S	464

### 2.2 Experimental setup

**Fig. 1:**
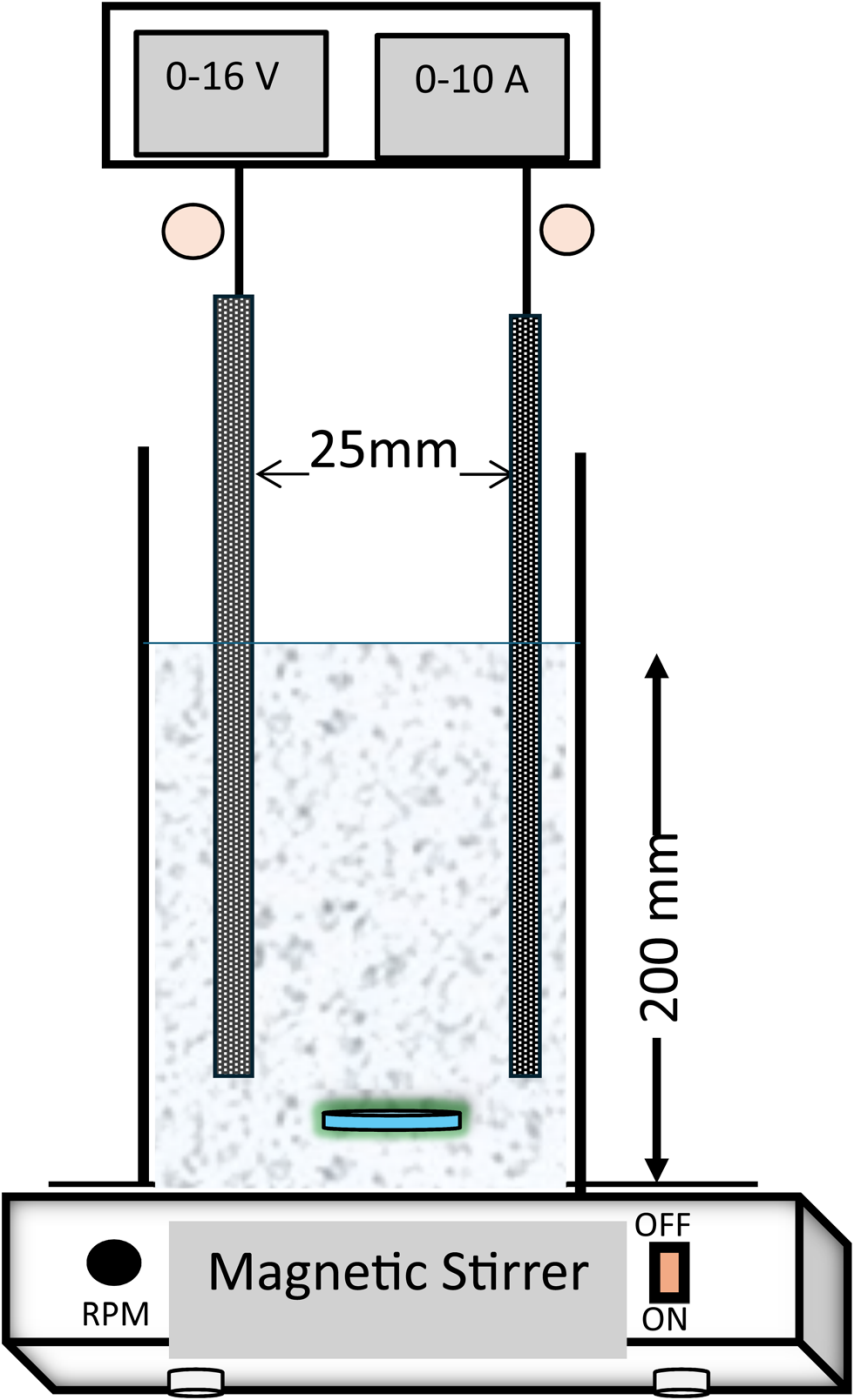
Experimental setup for electrocoagulation treatment.

The experiments were carried out in a custom-fabricated plexiglass reactor. The dimensions of the reactor were 20 cm (height) × 7 cm × 4 cm with an effective reactor volume of 575 ml and interelectrode gap of 25 mm (Fig. 1). The same pair of electrodes were used as anode and cathode. Three types of electrodes *viz.* aluminium (Al), iron (Fe) and stainless-steel (SS) electrodes were used and compared in terms of removal efficiency. To maintain the required current density, a regulated DC power supply (Aplab, L-3210) was used.

### 2.3 Analytical procedure

The colour removal efficiency was obtained from the initial and final dye concentrations after the treatment. The concentration of the dyes was determined by taking the absorbance at their maximum wavelength (*λ*_max_). Calibration curves were prepared for each dye at *λ*_max_ to determine the unknown concentration as depicted in Fig. S2.† Colour removal efficiency, COD removal efficiency and specific energy consumption were calculated using eqn (1), (2) and (3), respectively.1
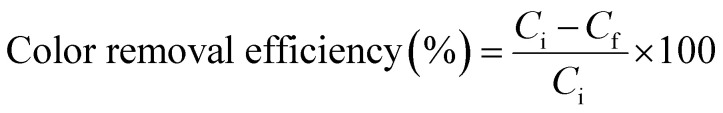
where *C*_i_ is the initial concentration of dye and *C*_f_ is the final concentration of dye.2
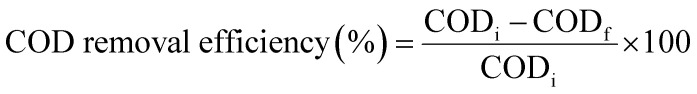
where COD_i_ is the initial COD of dye and *C*_f_ is the final COD of dye.3
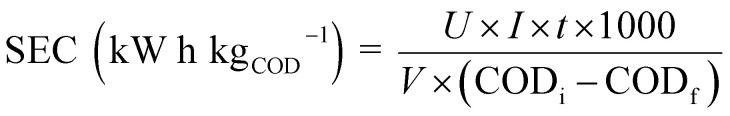
where *U* is the voltage (V), *I* is the current (A), *t* is the treatment time (h), *V* is the working volume of solution (L), and COD_i_ and COD_f_ are the initial and final COD (mg L^−1^), respectively.

The colour characteristics of the samples in terms of dominant wavelength, hue, luminance and purity before and after treatment were obtained using the spectrophotometric multi-wavelength method 2120-D.^18^

### 2.4 Optimal experimental design

In the present study, optimal experimental design was used to study the electrocoagulation removal of a mixture of azo dyes. I-optimal designs can also be referred to as integrated variance (IV) designs, and offer a lower average prediction variance across the entire region of the experiment. In RSM, I-optimality is preferred, where prediction is crucial. The algorithm picks points that minimize the integral of the prediction variance across the design space. For mixture experiments, I-optimal designs seem to be more appropriate as these minimize the average variance of prediction.^19^ Optimal design was used for seven variables (three dyes and four independent process variables) to maximize the colour and COD removal efficiency. This design was used to examine the treatment of a mixture of dyes *viz.* (i) methyl orange (ii) congo red and (iii) acid blue-113 for colour and COD removal efficiency under different process conditions. The range of the three mixture components was 0–100 mg L^−1^. The numeric variables were pH (5–9), current density (40–100 A m^−2^) and treatment time (10–30 min), and the categoric variable was electrode type (Al, Fe or SS). The ranges and levels of process variables are given in Table 2. Analysis of variance (ANOVA) statistics and model building were used to determine the significance of the model at *p* < 0.05 based on *F*-values and degrees of freedom. The goodness-of-fit was examined using the coefficient of determination (*R*^2^) and its closeness to the predicted *R*^2^. The *R*^2^ value demonstrates the extent to which the fitted model can explain the variation in the observed data. Adequate precision measures the signal-to-noise ratio (S/N), where an S/N > 4 is desirable. The fitted model is then generated both in coded terms and real components.

**Table 2:** Ranges and levels of independent process variables using I-optimal design for dye mixtures

Component	Dye	Units	Min	Max
*X*_1_	Methyl orange	mg L^−1^	0	100
*X*_2_	Congo red	mg L^−1^	0	100
*X*_3_	Acid blue-113	mg L^−1^	0	100

**Numeric variables (continuous)**				
*X*_4_	pH	—	5	9
*X*_5_	Current density	A m^−2^	40	100
*X*_6_	Treatment time	min	10	30

**Categoric variable (nominal)**				
*X*_7_	Electrode type	3 levels	Al [level 1]	
			Fe [level 2]	
			SS [level 3]	

## 3. Results and discussion

### 3.1 ANOVA modelling

ANOVA modelling was used for the statistical analyses of the response variables *viz.* colour and COD removal efficiency of the mixture of dyes. The optimal design comprised model terms (18 runs), lack of fit (5 runs) and replicates (5 runs), for a total of 28 experimental runs as given in Table 3.

**Table 3:** Optimal design matrix for three mixture variables, three continuous variables and one categorial variable along with experimental and predicted results using electrocoagulation treatment

Standard run	Build type	*X*_1_	*X*_2_	*X*_3_	*X*_4_	*X*_5_	*X*_6_	*X*_7_	*Y*_1_		*Y*_2_		*Y*_3_
		MO (mg L^−1^)	CR (mg L^−1^)	AB113 (mg L^−1^)	pH	CD (A m^−2^)	Time (min)	Electrode	Colour removal eff		COD removal eff		SEC (kW h kg_COD_^−1^)
									Actual (%)	Predicted (%)	Actual (%)	Predicted (%)	
1	Model	100	0	0	5.0	100	10.0	Al	38.6	38.4	8.6	9.1	171.5
2	Model	0	0	100	5.0	40	10.0	Al	97.8	97.4	75.8	74.4	4.6
3	Lack of fit	70.7	6.9	22.4	9.0	55.3	10.0	Al	31.9^a^	45.8	13.8	14.6	40.8
4	Model	0	100	0	9.0	100	10.0	Al	99.5	97.2	48.2	49.9	35.7
5	Model	0	100	0	5.0	40	30.0	Al	99.6	97.2	59.3	57.4	16.9
6	Model	100	0	0	9.0	40	30.0	Al	37.7^a^	38.6^a^	8.3	7.5	110.1
7	Lack of fit	23.9	7.5	68.6	6.1	75.7	30.0	Al	80.5	81.4	43.3	43.8	82.9
8	Model	0	0	100	9.0	100	30.0	Al	98.8	97.4	75.0	74.4	67.9
9	Model	100	0	0	5.0	40	10.0	Fe	74.6	76.0	31.4	34.1	7.4
10	Model	0	100	0	7.0	40	10.0	Fe	93.5	97.2	15.4	17.5	28.3
11	Replicate	0	100	0	7.0	40	10.0	Fe	96.2	97.2	19.2	17.5	20.9
12	Lack of fit	17.9	15.3	66.8	6.0	77.5	10.0	Fe	94.1	95.6	39.1	42.5	23.8
13	Model	100	0	0	9.0	100	10.0	Fe	94.5	94.9	47.2	41.3	23.9
14	Model	0	0	100	9.0	100	10.0	Fe	98.1	97.4	59.4	51.9	27.8
15	Model	0	0	100	5.0	40	30.0	Fe	93.8	97.4	48.5	51.9	18.5
16	Model	100	0	0	5.0	100	30.0	Fe	96.9	94.9	40.0	41.3	111.8
17	Model	0	100	0	7.0	100	30.0	Fe	96.8	97.2	26.6	29.1	220.5
18	Replicate	0	100	0	7.0	100	30.0	Fe	98.2	97.2	33.3	29.1	147.3
19	Model	0	100	0	5.0	100	10.0	SS	97.9	97.2	76.9	66.7	28.3
20	Model	100	0	0	7.0	70	20.0	SS	94.8	92.9	73.5	70.8	26.8
21	Replicate	100	0	0	7.0	70	20.0	SS	94.2	92.9	70.5	70.8	27.5
22	Model	0	0	100	5.0	100	20.0	SS	98.4	97.4	81.8	74.4	39.2
23	Replicate	0	0	100	5.0	100	20.0	SS	97.8	97.4	75.8	74.4	42.9
24	Model	0	0	100	9.0	40	20.0	SS	97.8	97.4	67.7	74.4	9.9
25	Replicate	0	0	100	9.0	40	20.0	SS	98.0	97.4	71.9	74.4	9.6
26	Lack of fit	2.0	98.0	0	5.0	77.5	25.2	SS	95.3	97.2	62.5	79.8	62.3
27	Model	0	100	0	9.0	40	30.0	SS	98.1	97.2	84.6	76.7	15.4
28	Lack of fit	100	0	0	9.0	100	30.0	SS	99.1	102.3	76.5	78.3	65.2

a Not used in ANOVA model building for colour removal efficiency (standard run no. 3 and 6).

#### 3.1.1 Colour removal efficiency

The highest colour removal efficiency of 99.6% was achieved for the congo red dye with aluminium electrodes, while the lowest colour removal efficiency was 31.9% for a dye mixture (70.7 mg L^−1^ methyl orange, 6.9 mg L^−1^ congo red and 22.4 mg L^−1^ acid blue-113) with aluminium electrodes (Table 3). ANOVA statistics for colour removal efficiency are given in Table S1a.† The best-fitted model was significant at 99.9% with an *F*-value of 91.1, and the lack-of-fit was also significant (not good for model prediction) at *p* = 0.0014. Hence, the model was reanalysed and two outliers were found (standard run 3 and 6 in Table 3) as shown in the normal residual plot (Fig. S3a†). These outliers were then ignored, and the ANOVA model was again generated for colour removal efficiency (Table S1b†). The fitted model was highly significant with a model *F*-value of 206 at a *p*-value < 0.0001 and a non-significant lack-of-fit (*p* = 0.053). The model summary statistics showed that the predicted *R*^2^ value of 0.936 is in reasonable agreement with the adjusted *R*^2^ value of 0.976. The model can explain 98.1% variance with a coefficient of variance of 2.1%. The adequate precision ratio (*S*/*N* ratio) is 69.15 (>4 is desirable), indicating that the chance of error due to noise is low for the fitted model. Thus, the model can be navigated in design space. The regression equation in coded units is given in eqn (4) and those in actual units are given in equations eqn (5)–(7).

4 *Y*_1_ = (69.12 × *X*_1_) + (97.20 × *X*_2_) + (97.37 × *X*_3_) + (9.42 × *X*_1_ × *X*_5_) − (40.12 × *X*_1_ × *X*_7_ [1]) + (16.32 × *X*_1_ × *X*_7_ [2]),

where *Y*_1_ = colour removal efficiency (%), *X*_1_ = methyl orange, *X*_2_ = congo red, *X*_3_ = acid blue-113, *X*_5_ = current density, *X*_7_ [1] = aluminium electrode, *X*_7_ [2] = iron electrode in coded units.

5 For Al electrode: *Y*_1_= (0.0701 × *X*_1_) + (0.9720 × *X*_2_) + (0.9736 × *X*_3_) + (0.00314 × *X*_1_ × *X*_5_),

6 For Fe electrode: *Y*_1_= (0.6346 × *X*_1_) + (0.9720 × *X*_2_) + (0.9736 × *X*_3_) + (0.00314 × *X*_1_ × *X*_5_),

7 For SS electrode: *Y*_1_= (0.7093 × *X*_1_) + (0.9720 × *X*_2_) + (0.9736 × *X*_3_) + (0.00314 × *X*_1_ × *X*_5_),

where *Y*_1_ = colour removal efficiency (%), *X*_1_ = methyl orange (mg L^−1^), *X*_2_ = congo red (mg L^−1^), *X*_3_ = acid blue-113 (mg L^−1^), *X*_5_ = current density (A m^−2^) in actual units.

The diagnostic plots were used for model validation; the normal percent probability plot of the response colour removal efficiency (Fig. S3b†) indicated that the design points are normally distributed. The actual *vs.* predicted plot (Fig. S3c†) showed that the model is suitable for predicting all responses. The OFAT plot showing the effect of pH, current density, treatment time and electrode type on the removal of a mixture of dyes (33.3 mg L^−1^ each) is depicted in Fig. S4.†

#### 3.1.2 COD removal efficiency

The stainless-steel electrodes achieved the best COD removal efficiency of 84.6% for Congo red, while the aluminium electrodes had the lowest COD removal efficiency of 8.3% for methyl orange (Table 3). The precision and adequacy of the best-fitted model were studied using the ANOVA table, which suggested the significance of the model at 99.9%, but lack-of-fit was also significant at a *p*-value of 0.0357, which is not good for the built model (Table S2†). The diagnostic plots were analyzed, and the Box–Cox plot suggested the log transformation of the response data (Fig. S5†). After log transformation, ANOVA model was built again, and the reduced best-fitted model was significant at *p* < 0.0001 (*F*-value = 75.9) with non-significant lack-of-fit (*p* = 0.354) (Table 4). The model summary statistics showed that the predicted *R*^2^ value of 0.931 is in close agreement with the adjusted *R*^2^ value of 0.968. The coefficient of variance (C. V.) is 3.2%, and the model explains 98.1% variance (*R*^2^ = 0.982), which confirms the high adequacy of the model proposed. The S/N ratio of 29.7 (more than 4) indicates that the chance of error due to noise is low for the fitted model. Thus, the model can be navigated in design space. The electrode type was found to be significant, whereas pH was found to be a non-significant variable in COD removal of all the dyes. Current density had a significant effect (two factor interaction) on the removal of both methyl orange and congo red. This suggests that the intensity of the electrical current applied during the treatment process plays a crucial role in degrading or removing these dyes. Treatment time was found to be significant only for congo red removal, but not for methyl orange and acid blue-113. This implies that congo red dye requires a longer duration of treatment to achieve effective removal, possibly due to its molecular structure, stability, or the mechanism of degradation. The transformed regression equation in coded units is given as eqn (8), whereas in actual units are given in eqn (9)–(11).

8 *Y*_2_ = (3.33 × *X*_1_) + (3.79 × *X*_2_) + (4.19 × *X*_3_) + (0.0982 × *X*_1_ × *X*_5_) − (1.22 × *X*_1_ × *X*_7_ [1]) + (0.293 × *X*_1_ × *X*_7_ [2]) + (0.092 × *X*_2_ × *X*_5_) + (0.162 × *X*_2_ × *X*_6_) + (0.188 × *X*_2_ × *X*_7_ [1]) − (0.6718 × *X*_2_ × *X*_7_ [2]) + (0.1215 × *X*_3_ × *X*_7_ [1]) − (0.2363 × *X*_3_ × *X*_7_ [2]),

where *Y*_2_ = COD removal efficiency (%), *X*_1_ = methyl orange, *X*_2_ = congo red, *X*_3_ = acid blue-113, *X*_5_ = current density, *X*_6_ = treatment time, *X*_7_ [1] = Al electrode, *X*_7_ [2] = Fe electrode in coded units.

9 For Al electrode: *Y*_2_ = (0.0188 × *X*_1_) + (0.0343 × *X*_2_) + (0.0431 × *X*_3_) + (0.00003 × *X*_1_ × *X*_5_) + (0.00003 × *X*_2_ × *X*_5_) + (0.00016 × *X*_2_ × *X*_6_),

10 For Fe electrode: *Y*_2_ = (0.0339 × *X*_1_) + (0.0257 × *X*_2_) + (0.0395 × *X*_3_) + (0.00003 × *X*_1_ × *X*_5_) + (0.00003 × *X*_2_ × *X*_5_) + (0.00016 × *X*_2_ × *X*_6_),

11 For SS electrode: *Y*_2_ = (0.0403 × *X*_1_) + (0.0373 × *X*_2_) + (0.0430 × *X*_3_) + (0.00003 × *X*_1_ × *X*_5_) + (0.00003 × *X*_2_ × *X*_5_) + (0.00016 × *X*_2_ × *X*_6_),

where *Y*_2_ = COD removal efficiency (%), *X*_1_ = methyl orange (mg L^−1^), *X*_2_ = congo red (mg L^−1^), *X*_3_ = acid blue-113 (mg L^−1^), *X*_5_ = current density (A m^−2^), *X*_6_ = treatment time (min), *X*_7_ [1] = Al electrode, *X*_7_ [2] = Fe electrode in actual units.

**Table 4:** ANOVA table and model statistics for COD removal efficiency after transformation

Source^a^	Sum of squares	Degrees of freedom	Mean sum of squares	*F*-Value	*p*-Value prob > *F*
Model	12.34	11	1.12	75.9	<0.0001*
Linear mixture	2.35	2	1.18	79.6	<0.0001*
*X*_1_ × *X*_4_	0.0524	1	0.052	3.55	0.0779#
*X*_1_ × *X*_7_	6.23	2	3.12	210.9	<0.0001*
*X*_2_ × *X*_4_	0.0677	1	0.067	4.59	0.0480*
*X*_2_ × *X*_6_	0.2146	1	0.214	14.5	0.0015*
*X*_2_ × *X*_7_	2.48	2	1.24	83.8	<0.0001
*X*_3_ × *X*_7_	0.2216	2	0.110	7.5	0.0050*
Lack of fit	0.1804	11	0.016	1.4	0.3538#

**Model statistics**					
Std deviation = 0.121, mean = 3.76, C.V. % = 3.23, PRESS = 0.865					
*R* ^2^ = 0.981, adjusted *R*^2^ = 0.968, predicted *R*^2^ = 0.931, adequate precision = 29.71					

a *X*_1_ = methyl orange, *X*_2_ = Congo red, *X*_3_ = acid blue 113, *X*_5_ = current density, *X*_6_ = treatment time, *X*_7_ = electrode, *significant at *p* ≤ 0.05, #not-significant at *p* ≤ 0.05.

**Fig. 2:**
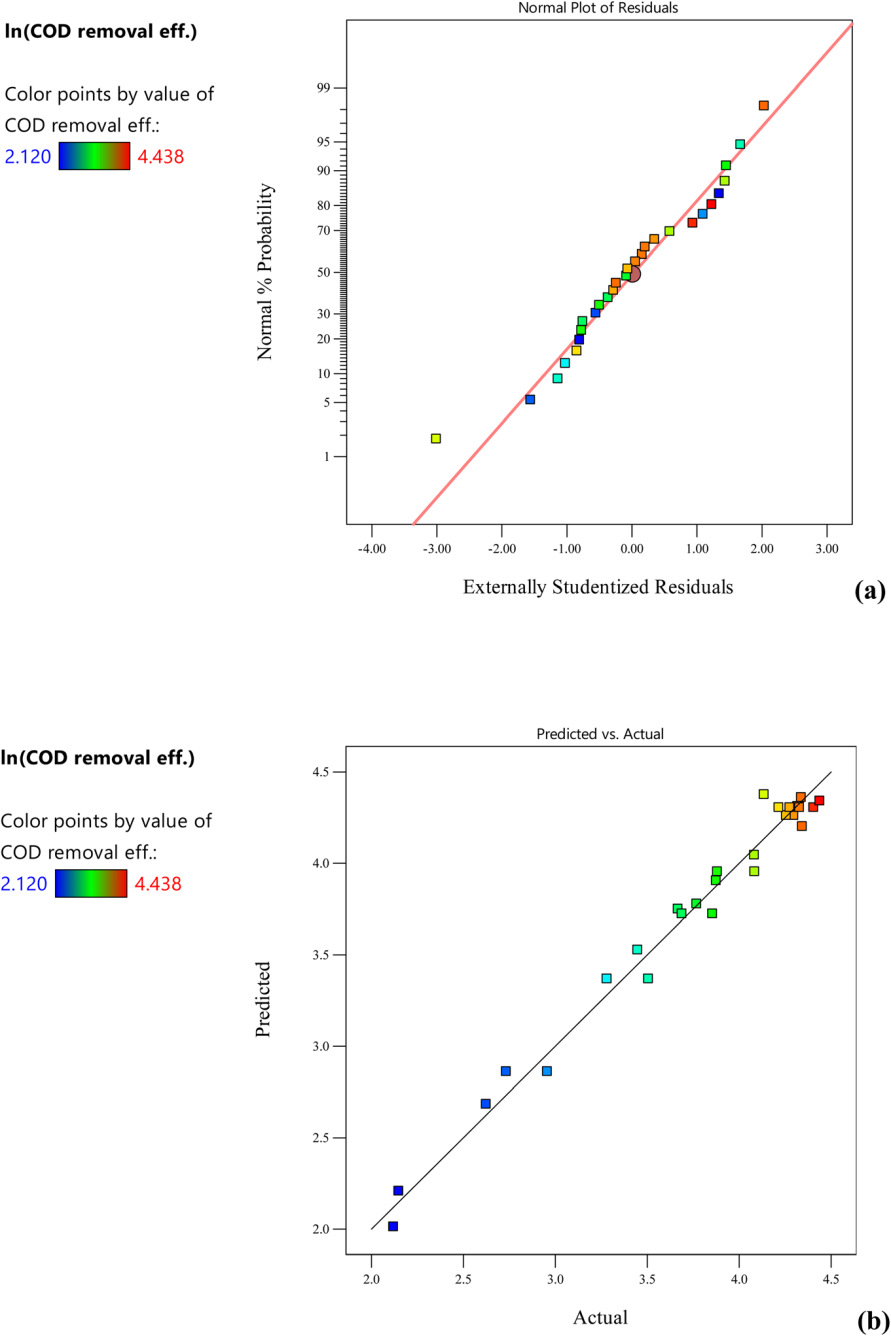
Normal percentage probability plot and (b) actual *vs.* predicted plot for COD removal efficiency of dye mixtures after log transformation.

Model suitability was tested from the diagnostic plots. The normal percentage probability plot for COD removal efficiency (Fig. 2a) explained the normal distribution of residual data. The actual *vs.* predicted plot (Fig. 2b) shows good agreement with predicted values and is in close proximity. Trace and overlay plots showing the effect of concentration of methyl orange, congo red and acid blue-113 using different electrode pairs on COD removal efficiency were also analyzed. In case of aluminium electrodes (Fig. S6a†), methyl orange and acid blue-113 have a more pronounced effect on COD removal efficiency than congo red concentration. High concentrations of acid blue-113 and congo red concentration are favourable for COD removal efficiency, whereas a high concentration of methyl orange negatively affects COD removal. The overlay plot showed that the aluminium electrodes can achieve the maximum COD removal and colour removal in the yellow region where the acid blue-113 concentration is higher (Fig. S6b†). The two-dimensional contour plot (Fig. S6c†) showed that the highest COD removal of 67.1% was obtained for acid blue-113 dye, whereas the lowest COD removal (10.5%) was achieved for methyl orange dye. The aluminium electrodes were not found to be suitable for the removal of methyl orange. The reason for the low removal efficiency might be the low adsorption capacity of aluminum oxide (Al_2_O_3_) for methyl orange due to the high initial COD (184 mg L^−1^) as compared to other two dyes *viz.* acid blue-113 (130 mg L^−1^) and congo red (96 mg L^−1^). An efficient colour removal effieincy (82%) for methyl orange (125 mg L^−1^) was achieved by Pi *et al.*^20^ using aluminium electrodes, but at a very high current density of 1850 A m^−2^ and high conductivity of 9.4 mS cm^−1^. To achieve high methyl orange removal by aluminium electrodes, high current density and conductivity are required. For iron electrodes, an increase in COD removal efficiency was observed at the higher acid blue-113 concentration and lower Congo red concentration, whereas methyl orange did not affect the COD removal (Fig. S7a†). The working region to achieve maximum COD and colour removal is at higher acid blue-113 concentrations (Fig. S7b†). The 2-D contour plot (Fig. S7c†) showed that the highest COD removal of 48.6% was obtained for acid blue-113 dye, whereas the lowest COD removal (20.9%) was obtained for Congo red dye.

**Fig. 3:**
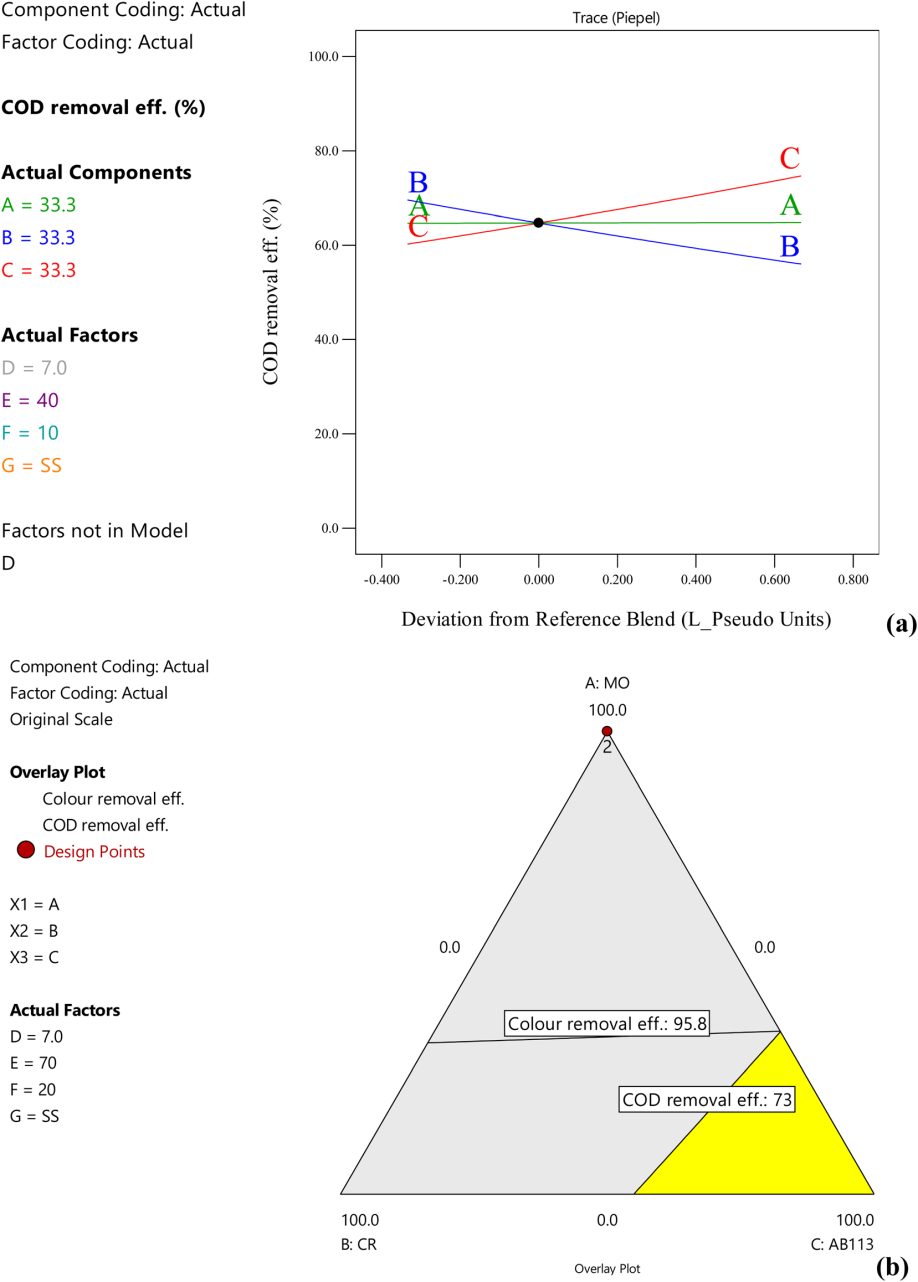
Effect of dye concentrations on COD removal efficiency of dye mixtures with stainless steel electrodes at pH = 7, 70 A m^−2^ and 20 min treatment time using (a) trace plots and (b) overlay plots (A, B, C = dye concentration, D = pH, E = current density, F = treatment time, G = electrode pair).

In the case of SS electrode pair, increasing methyl orange concentration did not significantly affect the COD removal. However, COD removal efficiency increases at higher concentrations of acid blue-113 and low congo red concentrations (Fig. 3a). Stainless steel is the most efficient among all the electrodes and is able to achieve 91% and 70% colour and COD removal efficiency even at 40 A m^−2^ for acid blue-113. The colour removal and COD removal were increased to 96% and 73% with the increase in current density to 70 A m^−2^ (Fig. 3b). At 100 A m^−2^, colour and COD removal efficiency increased to 98% and 78%, respectively, for methyl orange and Congo red in 20 min treatment time. Increases in the removal efficiency of dyes with increasing current density using stainless steel electrodes have been observed by many researchers.^21–24^ Increasing the current density increases the hydroxide flocs production, which is responsible for the adsorption of dye molecules due to the activation of redox reactions on the electrode surface.^22^ The 2-dimensional contour plot showed that the SS electrode pair was highly successful in COD removal of all the dyes with COD removal efficiencies ranging from 58.3% to 72.2%, where the highest COD removal (72.2%) was achieved for acid blue-113 dye (Fig. S8†). In the optimization process, the maximum colour removal of 99% and COD removal efficiency of 81.9% were obtained for a mixture of the dyes (33.3 mg L^−1^ each) under the optimal values of pH = 5.3, 100 A m^−2^, and treatment time of 30 min using SS electrodes, with 87 kW h kg^−1^ COD SEC.

In earlier studies, a colour removal efficiency of 55–100% and COD removal efficiency of 63–82% were achieved for different mixtures of dyes over a wide current density range of 55–1000 A m^−2^ with different electrode pairs (Table S3†). Moneer *et al.*^25^ achieved more than 86% colour removal efficiency for an acid green-20 and reactive yellow-17 mixture with aluminium electrodes at the highest current density (1000 A m^−2^) and a longer treatment time of 60 min, which contributed to obtaining a higher colour removal. A colour removal efficiency of 80% was obtained for a mixture of dyes (*n* = 6) at a higher current density of 400 A m^−2^ and 45 min treatment time using iron electrodes.^26^ Marquez *et al.*^27^ achieved only 55% colour removal and 67.5% COD removal of a complex mixture of tannery dyes (acid blue-113, acid blue-29 and brilliant green) at 220 A m^−2^ in 35 min, and further treatment was done with an electro-Fenton process to achieve higher removal efficiencies. In an earlier study by Kabdasli *et al.*,^28^ complete colour removal of a dye mixture was obtained with SS electrodes at a relatively high current density of 220 A m^−2^ and alkaline pH of 11.5. Under these conditions, a COD removal efficiency of only 63% was achieved for the dye mixture. The reason for the low COD removal efficiency could be the alkaline pH, which might be not favourable for the removal of the mixture of dyes. Electrocoagulation using iron electrodes obtained 85% colour removal efficiency at 108 A m^−2^ and 10 min treatment time for a binary mixture of blue disperse and yellow basic dyes.^29^ Keskin *et al.*^30^ achieved equivalent colour and COD removal efficiencies at a lower current density of 55 A m^−2^ in 10 min using iron electrodes for a binary mixture of reactive yellow and acid violet. However, iron electrodes have the disadvantage of rusting and holding residual iron, which gives the treated water a yellowish hue.

### 3.2 Colour characteristics of treated dye mixture

**Fig. 4:**
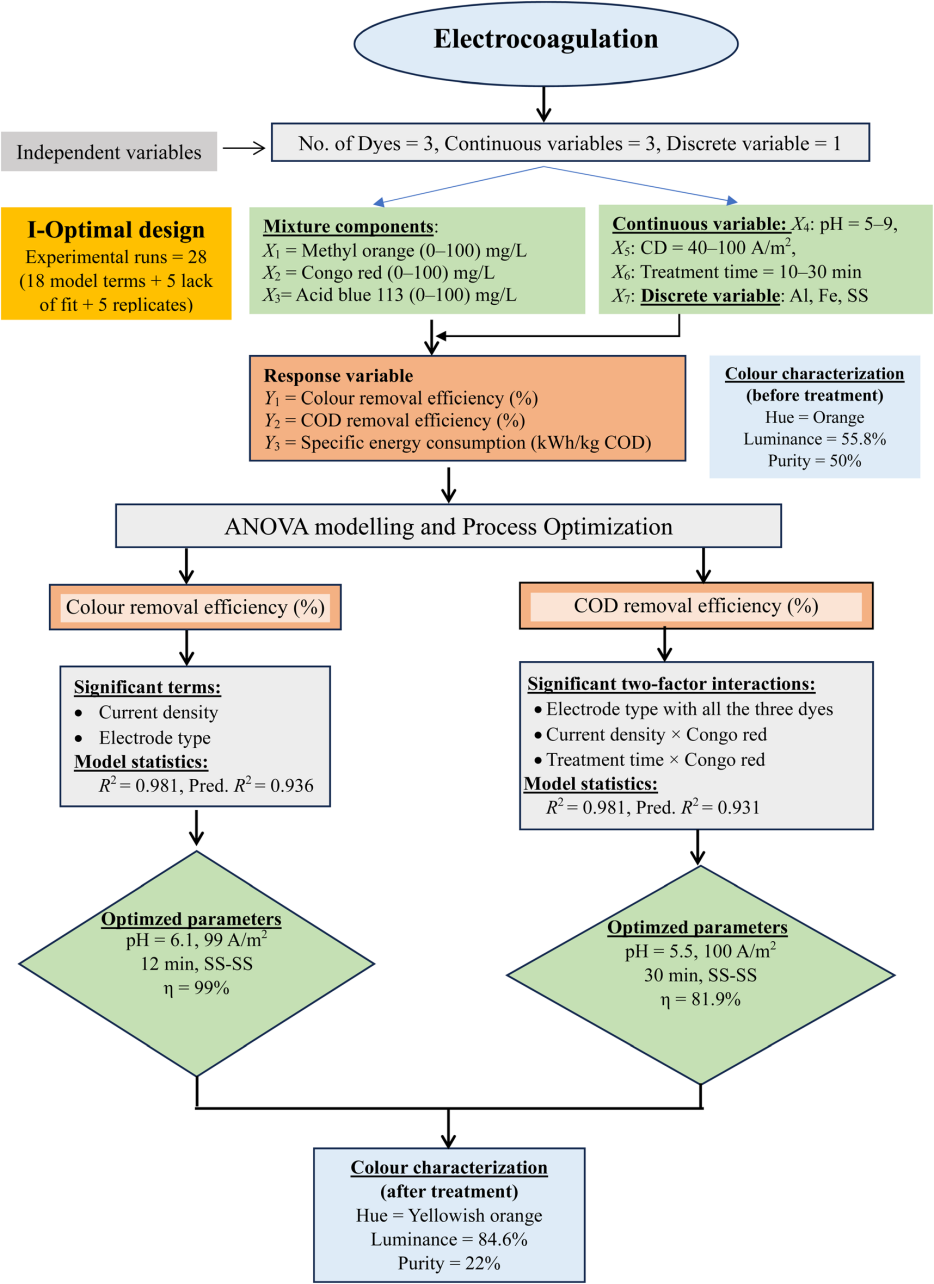
Flow chart for electrocoagulation treatment of ternary dye mixture.

The stainless-steel electrode pair was used to treat a mixture of dyes with 2 mg L^−1^ methyl orange and 98 mg L^−1^ congo red at pH = 5 and a current density of 78 A m^−2^. The absorption spectra before and after treatment (25 min) are shown in Fig. S9.† Characteristic peaks at 346 nm and 497 nm were observed, which are similar to those of congo red dye due to the high concentration of congo red in the mixture. Both peaks were removed to a great extent, which indicated the effective removal of the dye mixture using SS electrode pair without the formation of any intermediate. The dominant wavelength of the untreated dye mixture was 590 nm with an orange hue, 55.8% luminance and 50% purity (Table 5). Although a marginal decrease in the dominant wavelength was observed, significant improvements in purity (22%) and luminance (84.6%) were observed in the treated sample. In the present study, stainless-steel electrode pair was found to be best for the treatment of a mixture of methyl orange, congo red and acid blue-113 among all the electrode pairs. A flow chart of the work carried out is shown in Fig. 4.

**Table 5:** Colour characteristics of dye mixtures for electrocoagulation treatment using stainless-steel electrode pair

Colour characteristic	Unit	Before treatment	After treatment
Dominant wavelength	nm	590	582
Hue	—	Orange	Yellowish orange
Luminance	%	55.8	84.6
Purity	%	50	22

## 4 Meta-analysis of electrocoagulation treatment of dyes

Meta-analysis of earlier research studies was done to propose the best operating conditions for the treatment of dyes *via* electrocoagulation using three types of electrodes *viz*. aluminium, stainless-steel and iron. The optimum process conditions for the removal of dyes *via* electrocoagulation using the different electrodes are given in Table S4.†Table 6 shows four process factors *viz.* dye concentration, pH, current density and treatment time with colour removal efficiency as the response variable for three different electrode pairs.

**Table 6:** Range of current density and treatment time along with colour removal efficiency for electrocoagulation dye removal using different electrode pairs

Electrode pair	Current density (A m^−2^)	Treatment time (min)	Colour removal efficiency (%)	Dyes	Reference
Al–Al	50–200	5–30	98.0–99.4	Methylene blue	31
				Disperse blue 56	32
Al–Al	230–300	5–40	81.0–99.5	Acid blue-113	33
				Acid orange-7	34 and 37
				Acid red-336	35
				Acid red-18	36
SS–SS	110	40	99.8	Reactive orange-84	21
SS–SS	60–72	20–30	90–100	Rhodamine-B	23
				Brilliant green	24
Fe–Fe	30–80	12–120	74.0–99.9	Methylene blue	40
				Reactive red-196	41
				Methyl orange	42
				Indigo blue	43
Fe–Fe	100–250	5–60	80.9–99.6	Reactive orange-84	21
				Congo red	44
				Reactive red-24	45
				Reactive black-5	46
				Disperse red-74	47
				Methyl orange	48
Fe–Fe	300–350	4–20	90.7–98.6	Disperse brown S-3R	49
				Alizarin yellow	50
				Methyl orange	50
				Acid orange-7	51

### 4.1 Aluminium electrode

Liu and Wu^31^ and Dassa *et al.*^32^ utilised the lowest current density range (50–200 A m^−2^), resulting in colour removal efficiencies of 99.4% and 98% for methylene blue and disperse blue-56, respectively, under these conditions. The low initial dye concentrations (25–30 mg L^−1^) may have contributed to the high removal efficiency for methylene blue and dispersion blue-56 at low current densities. In the current density range of 230–300 A m^−2^, five studies^33–37^ achieved colour removal efficiencies of 81–99.5% for acid blue-113, acid orange-7, acid red-18 and acid red-336 dyes. An increase in the utilisation of current density was observed for higher dye concentrations to achieve efficient removal efficiency. However, for acid orange-7, only 81% colour removal efficiency was achieved at 229 A m^−2^ by Taheri,^37^ which showed that for acid orange-7, a high current density is required. The highest current densities were used by Pi *et al.*^20^ and Ramya Sankar and Sivasubramanian^38^ for methyl orange and congo red dye removal, respectively. About 97% colour removal efficiency was achieved for methyl orange dye at 1850 A m^−2^. This showed that aluminium electrodes are not suitable for treating methyl orange at low current density. Electrocoagulation removal of congo red gave 89.3% colour removal efficiency at a significant current density (2040 A m^−2^) using RSM. The reason for this high current density might be the alkaline pH of 9.5. At pH > 9, Al(OH)_4_ is produced, which causes the reduction of flocs and restoration of the adsorbed colourant, leading to a decrease in removal efficiency.^39^

### 4.2 Stainless-steel electrode

Adeogun and Balakrishnan^23^ and Marquez *et al.*^24^ used the lowest current densities, in the range of 60–72 A m^−2^, and achieved a colour removal efficiency of more than 90%. Adeogun and Balakrishnan^23^ obtained 90% colour removal efficiency for 50 mg per L rhodamine-B at 72 A m^−2^, whereas Marquez *et al.*^24^ achieved complete decolourisation of 338 mg per L brilliant green at the lowest current density of 60 A m^−2^. The reason for the different colour removal efficiencies under comparable current densities might be the differences in the molecular structures and dye classes. Rhodamine-B is a xanthene dye, whereas brilliant green is a triphenylmethane dye, which might be responsible for the different removal efficiencies under similar process conditions. For reactive orange-84, a current density of 110 A m^−2^ and treatment time of 40 min were used to achieve 99.8% colour removal efficiency.^21^

### 4.3 Iron electrode

Five studies^5,40–43^ achieved colour removal efficiency between (74–99.9)%. For methyl orange, indigo blue and reactive red-196, more than 95% colour removal efficiency was obtained at a low current density of 30–62 A m^−2^. The reason for the high removal efficiency of these dyes could be the low dye concentration and long treatment times (40–120 min). However, for methyl orange, only 74% colour removal efficiency was achieved at ∼60 A m^−2^ for a dye concentration of 15 mg L^−1^. This showed that the iron electrode is not an effective electrode for methyl orange treatment at 60 A m^−2^ of current density. Similarly, 80% methylene blue (thiazine class) dye removal efficiency was obtained at 80 A m^−2^ using iron electrodes. Six studies^21,44–48^ achieved colour removal efficiencies in the range of 80.9–99.6% in the high current density range of 100–250 A m^−2^. More than 89% removal efficiency was obtained for the dyes reactive red-24, methyl orange, disperse red-74 and Congo red at 100–150 A m^−2^. Mook *et al.*^46^ used a higher current density of 250 A m^−2^ and 50 min of treatment time for the electrocoagulation treatment of reactive black-5 and obtained 80.9% colour removal efficiency. Colour removal efficiencies in the range of 90.7–98.6% were obtained for disperse brown,^49^ alizarin yellow, methyl orange^50^ and acid orange-7 (ref. 51) in the high current density range of 300–350 A m^−2^. In a nutshell, increasing the current density increases the colour removal efficiency with shorter treatment time.

**Fig. 5:**
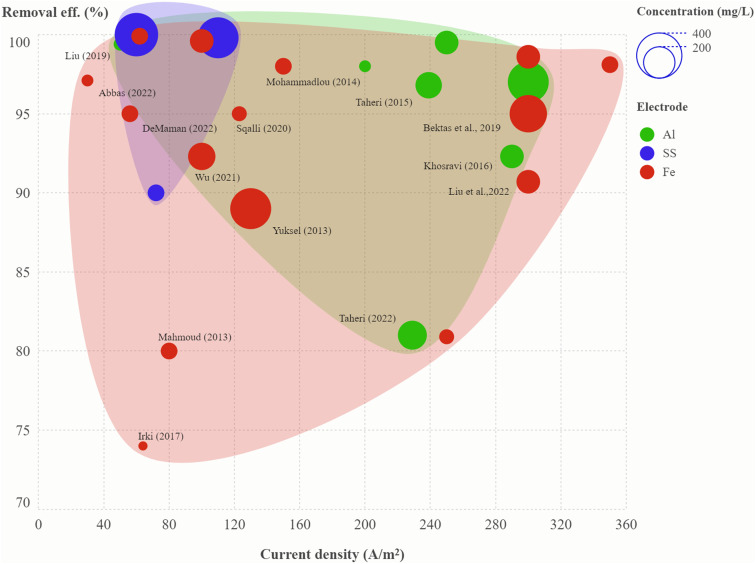
Convex hull graph showing effect of current density on colour removal efficiency. Size of the circle represents dye concentration and colour of the circle represents the electrode type. Circles are labelled with the corresponding reference (details in Table S4†).

A convex hull graph showing the effect of current density on colour removal efficiency, in which the size of the circle represents dye concentration and the colour of the circle represents the electrode type is presented in Fig. 5; the circles are labelled with the relevant references. In another view of the convex hull graph showing the effect of pH on colour removal efficiency, the size of the circle represents treatment time and the colour of the circle represents the electrode type, and the circles are labelled with the dye name (Fig. S10†). The aluminium electrodes were shown to be efficient in the treatment of most dyes, with colour removal rates ranging from 81–99.5% at current densities of 50–300 A m^−2^ and treatment times of 5–40 min, while treatment of methyl orange and congo red with aluminium electrodes was not effective at low current density (Table 6). The stainless-steel electrode pair was most effective for brilliant green, rhodamine-B and reactive orange-84 at a current density of 60–110 A m^−2^ and treatment time of 20–40 min. Electrocoagulation treatment using the iron electrode pair was found to be effective for indigo blue, methylene blue, methyl orange and reactive red-196.

## 5. Conclusions

A maximum COD removal efficiency of 81.9% was achieved for mixture of dyes (∼33.3 mg L^−1^ each) at the highest level of current density (100 A m^−2^) and treatment time (30 min) using a stainless-steel electrode pair. The pH of the solution may be kept in the working range of pH = 5–9, as it is not a model term in the regression equation. Meta-analysis showed that the current density range of 50–300 A m^−2^ was effective in colour removal for most of the dyes except methyl orange and congo red using aluminium electrode. Stainless-steel electrode pair was established to be the most efficient as compared to aluminium and iron electrode pairs for achieving greater than 90% colour removal efficiency at a current density of 60–110 A m^−2^ and 20–40 min. of treatment time. Convex hull plots gave interesting insights related to a significantly higher number of studies using aluminium electrodes followed by iron electrodes. Stainless-steel electrodes gave higher colour removal efficiency but at higher initial current density. An optimal pH range of 6.0–8.0 is most prevalent for iron and stainless-steel electrode pair, whereas an acidic pH between 4.0–6.0 is most commonly used in electrocoagulation treatment using aluminium electrode pair. The strength of this study lies in its evaluation of a ternary mixture of dyes and colour characterization using spectrophotometric analysis. This is the first study of this kind in which two dozen dyes have been grouped together in an advanced scatter plot by binding categorical variables (electrode type) by colour code and representing a another variable (current density) by the size of the circle to give a visual distribution of the data.

## Data availability

Data is available on request. Data related to meta-analysis can be checked from the respective papers.

## Author contributions

M. S. B.: conceptualization, methodology, review, visualization and supervision, A. S.: experimentation, methodology, writing-original draft.

## Conflicts of interest

Authors declare no conflict of interest.

## Supplementary Material

RA-015-D4RA08485C-s001

## References

[cit1] Ali H. (2010). Biodegradation of synthetic dyes—a review. Water, Air, Soil Pollut..

[cit2] Singh K., Arora S. (2011). Removal of synthetic textile dyes from wastewaters: a critical review on present treatment technologies. Crit. Rev. Environ. Sci. Technol..

[cit3] Manikandan P., Palanisamy P. N., Baskar R., Sivakumar P., Sakthisharmila P. (2016). Optimization of treatment efficiency of UV/H_2_O_2_ process on simulated textile industry wastewater. Desalin. Water Treat..

[cit4] Zafar A. M., Naeem A., Minhas M. A., Hasan M. J., Rafique S., Ikhlaq A. (2024). Removal of reactive dyes from textile industrial effluent using electrocoagulation in different parametric conditions of aluminium electrodes. Total Environ. Adv..

[cit5] Abbas S. H., Younis Y. M., Rashid K. H., Khadom A. A. (2022). Removal of methyl orange dye from simulated wastewater by electrocoagulation technique using Taguchi method: kinetics and optimization approaches. React. Kinet., Mech. Catal..

[cit6] Hussain S., Yaqub A., Bhatti Z. A., Khan R., Ajab H., Isa M. H. (2023). Electrocoagulation of reactive orange 16 textile dye solution using steel, aluminum, and copper metal plates as electrodes. Surf. Eng. Appl. Electrochem..

[cit7] Husein S., Dewi E. L. (2024). The impact of sodium chloride (NaCl) concentrations on electrocoagulation for simultaneous tartrazine dye removal and hydrogen production. Eng. Proc..

[cit8] Sharma S., Sharma S. K., Acharya S., Khandegar V. (2024). Feasibility and sludge analysis of electrocoagulation process for direct violet-35 dye remediation. Waste Manag. Bull..

[cit9] Sugha A., Gilhotra V., Bhatti M. S. (2025). Electrocoagulation and anodic oxidation for the treatment of commercial dyes and real textile effluent: Meta-analysis for optimal operating conditions. Water Conserv. Sci. Eng..

[cit10] Sezer M., Goktas C. G., Isgoren M., Veli S., Bingol S. N., Cakmak I. N. (2024). Response surface optimization of electrocoagulation for the removal of CI disperse red 343 and isolan bordeaux 2S-B dyes. Desalin. Water Treat..

[cit11] Gautam K., Kumar Y., Sonawane S., Kumar S. (2025). Electrochemical treatment of wastewater containing reactive blue 4 (RB 4) dye: RSM and ANN optimization, techno economic analysis and sludge characterization. Clean Chem. Eng..

[cit12] Lamhar R., Kambuyi T. N., Kherbeche A., Zmirli Z., Bejjany B., Aguelmous A., Digua K., Dani A. (2025). Foam investigation and optimization by response surface methodology of electrocoagulation process for textile wastewater decolorization in single-channel reactor. Chem. Eng. Res. Des..

[cit13] Canan Pekel L., Ertunc S., Zeybek Z., Alpbaz M. (2013). Optimization of electrochemical treatment of textile dye wastewater. Manag. Environ. Qual. Int. J..

[cit14] Moulai-Mostefa N., Ladjelat S., Kermet-Said H., Krea M., Tir M. (2014). Optimization of operational parameters in the pretreatment of surface water by electrocoagulation using a response surface method. Desalin. Water Treat..

[cit15] Singh H., Singh G., Bhatti M. S., Reddy A. S. (2015). Textile dyebath wastewater decolorization by electrolytic processes: response surface optimization using IV-optimal design. Desalin. Water Treat..

[cit16] Tamne G. B., Nanseu-Njiki C. P., Bodoki E., Săndulescu R., Oprean R., Ngameni E. (2016). Removal of nitroaniline from water/ethanol by electrocoagulation using Response Surface Methodology. Clean:Soil, Air, Water.

[cit17] Eryürük K., Eryürük Ş., Un U. T., Ogutveren U. B. (2021). A design of experiment approach of cattle slaughterhouse wastewater treatment by electrocoagulation method. Desalin. Water Treat..

[cit18] APHA , Standard Methods for the Examination of Water and Wastewater, American Public Health Association, Washington, DC, 23rd edn, 2017

[cit19] Goos P., Jones B., Syafitri U. (2016). I-optimal design of mixture experiments. J. Am. Stat. Assoc..

[cit20] Pi K. W., Xiao Q., Zhang H. Q., Xia M., Gerson A. R. (2014). Decolorization of synthetic methyl orange wastewater by electrocoagulation with periodic reversal of electrodes and optimization by RSM. Process Saf. Environ. Prot..

[cit21] Yuksel E., Eyvaz M., Gurbulak E. (2013). Electrochemical treatment of colour index reactive orange 84 and textile wastewater by using stainless steel and iron electrodes. Environ. Prog. Sustainable Energy.

[cit22] Vianney M. J., Muthukumar K. (2016). Studies on dye decolorization by ultrasound assisted electrocoagulation. Clean:Soil, Air, Water.

[cit23] Adeogun A. I., Balakrishnan R. B. (2017). Kinetics, isothermal and thermodynamics studies of electrocoagulation removal of basic dye rhodamine B from aqueous solution using steel electrodes. Appl. Water Sci..

[cit24] Marquez A. A., Coreno O., Nava J. L. (2022). Removal of brilliant green tannery dye by electrocoagulation. J. Electroanal. Chem..

[cit25] Moneer A. A., El-Mallah N. M., Ramadan M. S., Shaker A. M. (2021). Removal of Acid Green 20 and Reactive Yellow 17 dyes by aluminum electrocoagulation technique in a single and a binary dye system. Egypt. J. Aquat. Res..

[cit26] Balla W., Essadki A. H., Gourich B., Dassaa A., Chenik H., Azzi M. (2010). Electrocoagulation/electroflotation of reactive, disperse and mixture dyes in an external-loop airlift reactor. J. Hazard. Mater..

[cit27] Márquez A. A., Coreño O., Nava J. L. (2023). Abatement of a complex mixture of dyes in the presence of chlorides by electrocoagulation and active chlorine-based photoelectro-Fenton-like processes. Process Saf. Environ. Prot..

[cit28] Kabdaşlı I., Vardar B., Arslan-Alaton I., Tünay O. (2009). Effect of dye auxiliaries on color and COD removal from simulated reactive dyebath effluent by electrocoagulation. Chem. Eng. J..

[cit29] Ayhan Şengil İ., Özdemir A. (2012). Simultaneous decolorization of binary mixture of blue disperse and yellow basic dyes by electrocoagulation. Desalin. Water Treat..

[cit30] Keskin C. S., Özdemir A., Şengil İ. A. (2011). Simultaneous decolorization of binary mixture of reactive yellow and acid violet from wastewaters by electrocoagulation. Water Sci. Technol..

[cit31] Liu N., Wu Y. (2019). Removal of methylene blue by electrocoagulation: a study of the effect of operational parameters and mechanism. Ionics.

[cit32] Dassaa A., Wakrim A., Byoud F., Saib N., Cherif A., Essadki A. H., Azzi M. (2021). Electrochemical degradation of disperse blue 56 from aqueous solution using aluminum electrodes. Mater. Today: Proc..

[cit33] Taheri M., Moghaddam M. R., Arami M. (2014). A comparative study on removal of four types of acid azo dyes using electrocoagulation process. Environ. Eng. Manage. J..

[cit34] Taheri M., Moghaddam M. R., Arami M. (2015). Improvement of the/Taguchi/design optimization using artificial intelligence in three acid azo dyes removal by electrocoagulation. Environ. Prog. Sustainable Energy.

[cit35] Amour A., Merzouk B., Leclerc J. P., Lapicque F. (2016). Removal of reactive textile dye from aqueous solutions by electrocoagulation in a continuous cell. Desalin. Water Treat..

[cit36] Khosravi R., Hazrati S., Fazlzadeh M. (2016). Decolorization of AR18 dye solution by electrocoagulation: sludge production and electrode loss in different current densities. Desalin. Water Treat..

[cit37] Taheri M. (2022). Techno-economical aspects of electrocoagulation optimization in three acid azo dyes' removal comparison. Clean Chem. Eng..

[cit38] Ramya Sankar M. S., Sivasubramanian V. (2020). Application of statistical design to optimize the electrocoagulation of synthetic congo red dye solution and predicting the mechanism. Int. J. Environ. Sci. Technol..

[cit39] Khorram A. G., Fallah N. (2018). Treatment of textile dyeing factory wastewater by electrocoagulation with low sludge settling time: optimization of operating parameters by RSM. J. Environ. Chem. Eng..

[cit40] Mahmoud M. S., Farah J. Y., Farrag T. E. (2013). Enhanced removal of methylene blue by electrocoagulation using iron electrodes. Egypt. J. Pet..

[cit41] Assadi A., Soudavari A., Mohammadian M. (2016). Comparison of electrocoagulation and chemical coagulation processes in removing reactive red 196 from aqueous solution. J. Hum., Environ., Health Promot..

[cit42] Irki S., Ghernaout D., Naceur M. W. (2017). Decolourization of methyl orange (MO) by electrocoagulation (EC) using iron electrodes under a magnetic field (MF). Desalin. Water Treat..

[cit43] De Maman R., Behling L., da Luz V. C., Dervanoski A., Rosa C. D., Pasquali G. D. (2022). Oxidation of textile dye through electrocoagulation process using scrap iron electrodes. Water, Air, Soil Pollut..

[cit44] Mohammadlou N., Rasoulifard M. H., Vahedpour M., Eskandarian M. R. (2014). The kinetic and thermodynamic study for decolorization of congo red from aqueous solution using electrocoagulation process. J. Appl. Chem. Res..

[cit45] Ghalwa N. A., Saqer A. M., Farhat N. B. (2016). Removal of reactive red 24 dye by clean electrocoagulation process using iron and aluminum electrodes. J. Chem. Eng. Process Technol..

[cit46] Mook W. T., Aroua M. K., Szlachta M., Lee C. S. (2017). Optimisation of reactive black 5 dye removal by electrocoagulation process using response surface methodology. Water Sci. Technol..

[cit47] Houssini N. S., Essadki A., ElQars E. (2020). The simultaneous removal of reactive and disperse dyes by electrocoagulation process with a bipolar connection of combined iron and aluminum electrodes: experimental design and kinetic studies. Mediterr. J. Chem..

[cit48] Wu Z., Dong J., Yao Y., Yang Y., Wei F. (2021). Continuous flowing electrocoagulation reactor for efficient removal of azo dyes: kinetic and isotherm studies of adsorption. Environ. Technol. Innovation.

[cit49] Bektas N., Ozden M., Tekbas M., Calıskan Y. (2019). Decolourisation of disperse brown dye solution by electrocoagulation process with Al and Fe electrodes. Gazi Univ. J. Sci..

[cit50] Liu Y., Li C., Bao J., Wang X., Yu W., Shao L. (2022). Degradation of azo dyes with different functional groups in simulated wastewater by electrocoagulation. Water.

[cit51] Chafi M., Gourich B., Essadki A. H., Vial C., Fabregat A. (2011). Comparison of electrocoagulation using iron and aluminium electrodes with chemical coagulation for the removal of a highly soluble acid dye. Desalination.

