# Supplementary material for: Optimization of electrocoagulation removal of a mixture of three azo dyes: spectrophotometric colour characteristics for best operating conditions
Source: RSC Adv. 2025 Feb 27;15(9):6492–505. doi: 10.1039/d4ra08485c (PMC11865942; doi:10.1039/d4ra08485c)
Supplement: RA-015-D4RA08485C-s001 [file RA-015-D4RA08485C-s001.pdf]

Supplementary Information (SI) for RSC Advances.
This journal is © The Royal Society of Chemistry 2025

SUPPLEMENTARY INFORMATION

Optimization of electrocoagulation removal of mixture of three azo dyes: Spectrophotometric colour characteristics for best operating conditions

Aditi Sugha and Manpreet Singh Bhatti*

Department of Botanical & Environmental Sciences, Guru Nanak Dev University, Amritsar, Punjab, India

asugha12@gmail.com (A Sugha); mbhatti.dobes@gndu.ac.in (M S Bhatti)

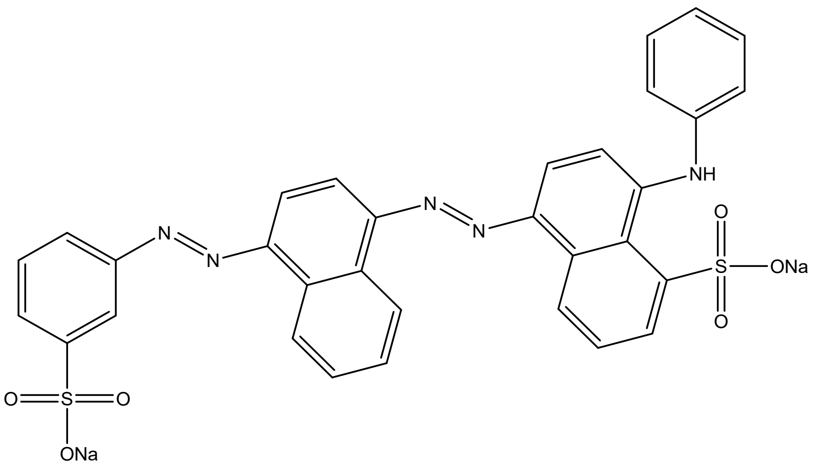

(a) Acid blue 113

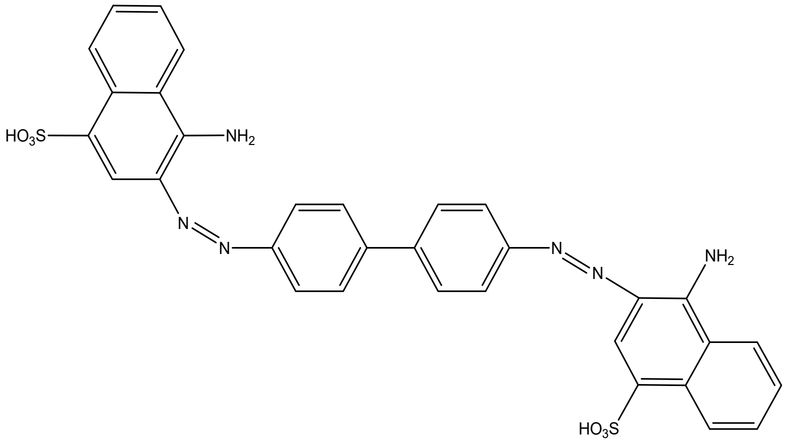

(b) Congo red

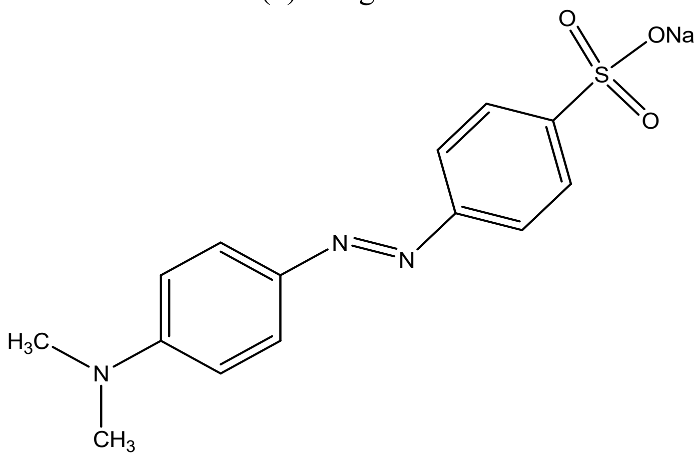

(c) Methyl orange

Fig. S1 Structure of dyes (a) Acid blue 113 (b) Congored (c) Methyl orange

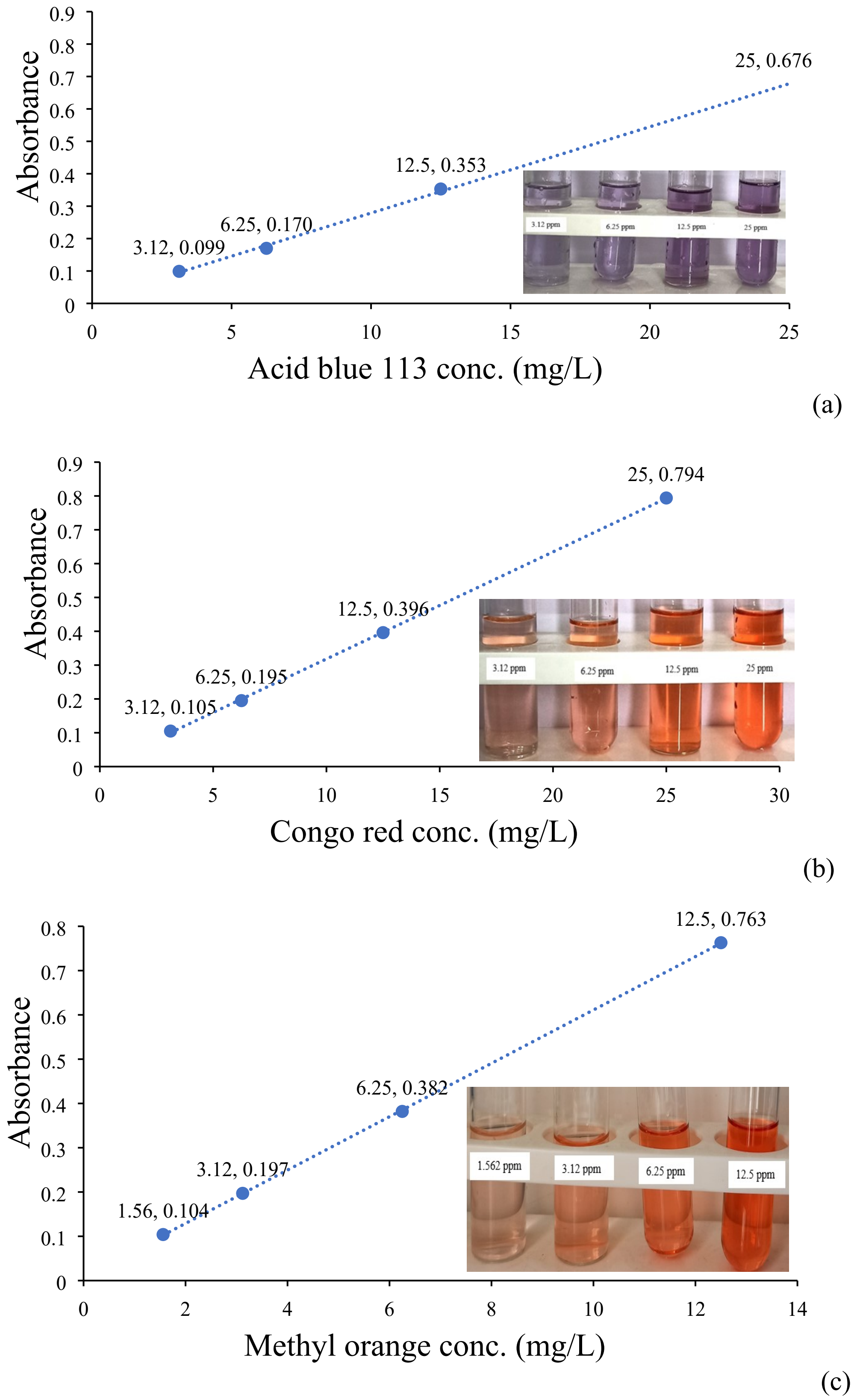

Fig. S2 Standard curve of dyes with colour intensity of prepared standards for (a) Acid blue-113 (b) Congo red (c) Methyl orange

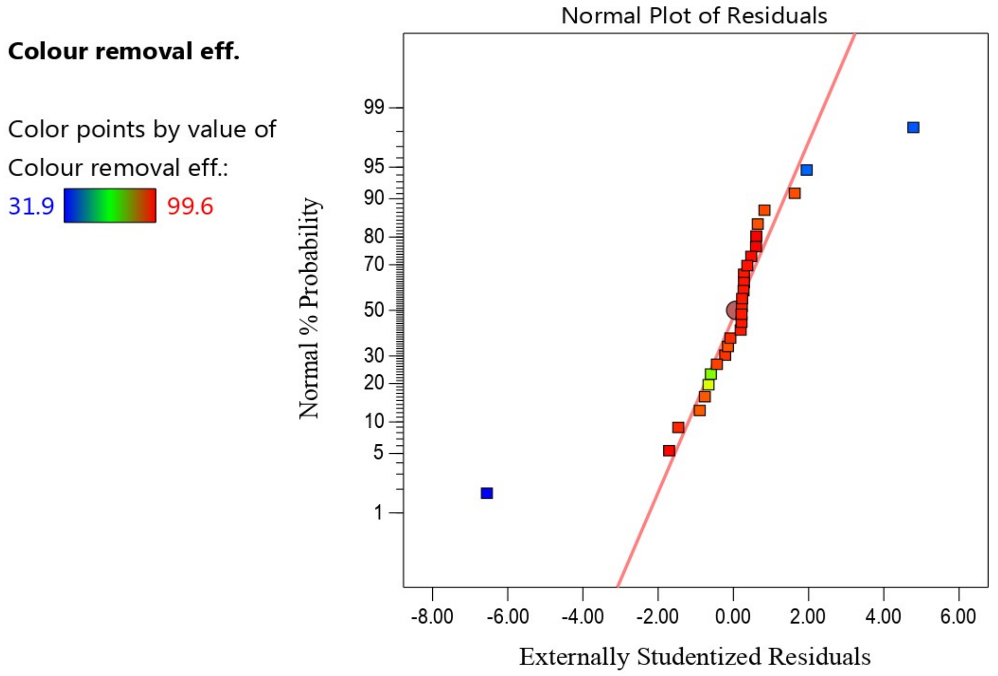

(a)

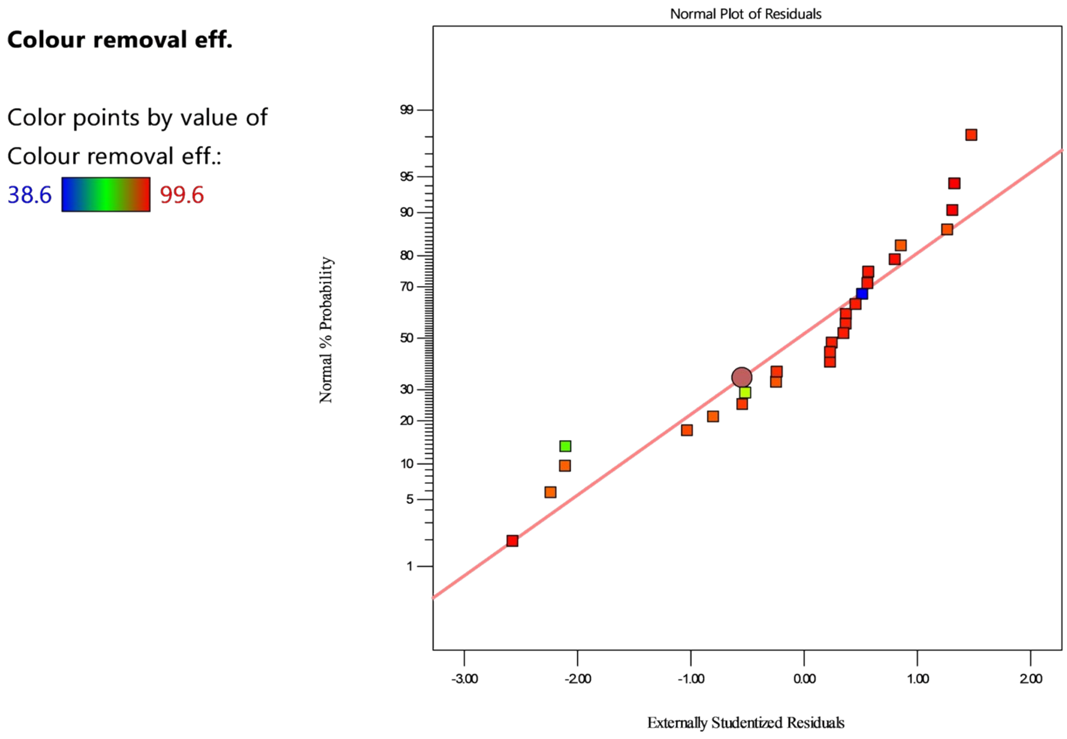

(b)

Fig. S3 Normal percentage probability plot of colour removal for dye mixtures using electrocoagulation (a) with outlier (b) after removing outliers

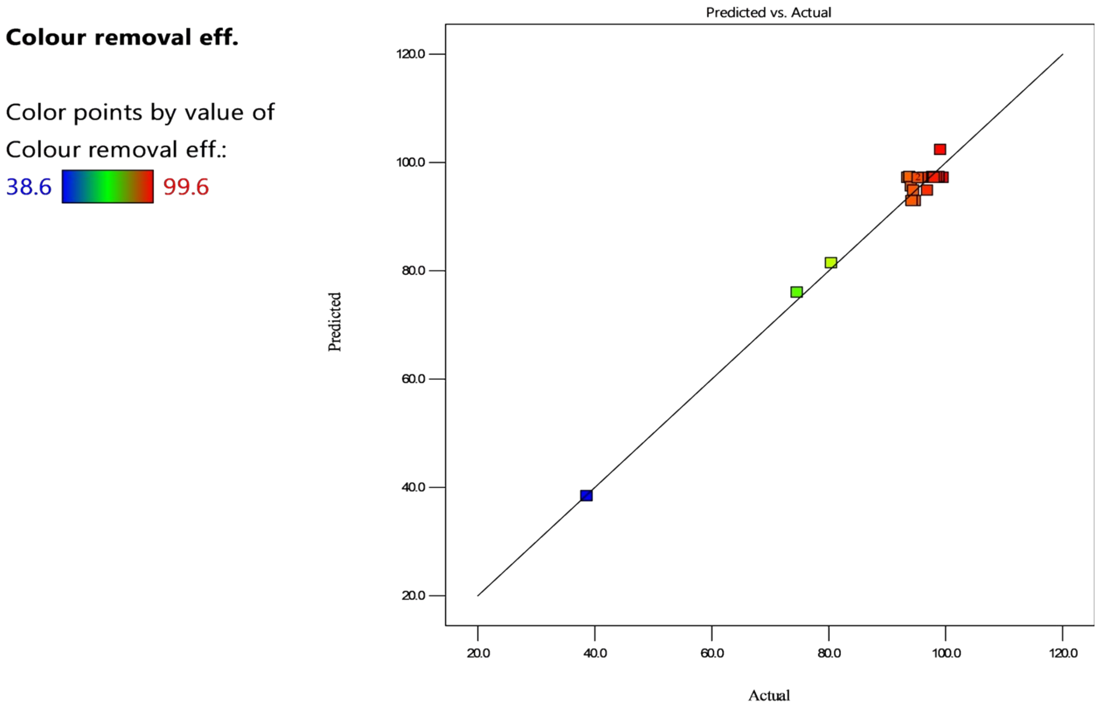

Fig. S3c Actual *vs*. predicted plot for colour removal efficiency for dye mixtures as per optimal design

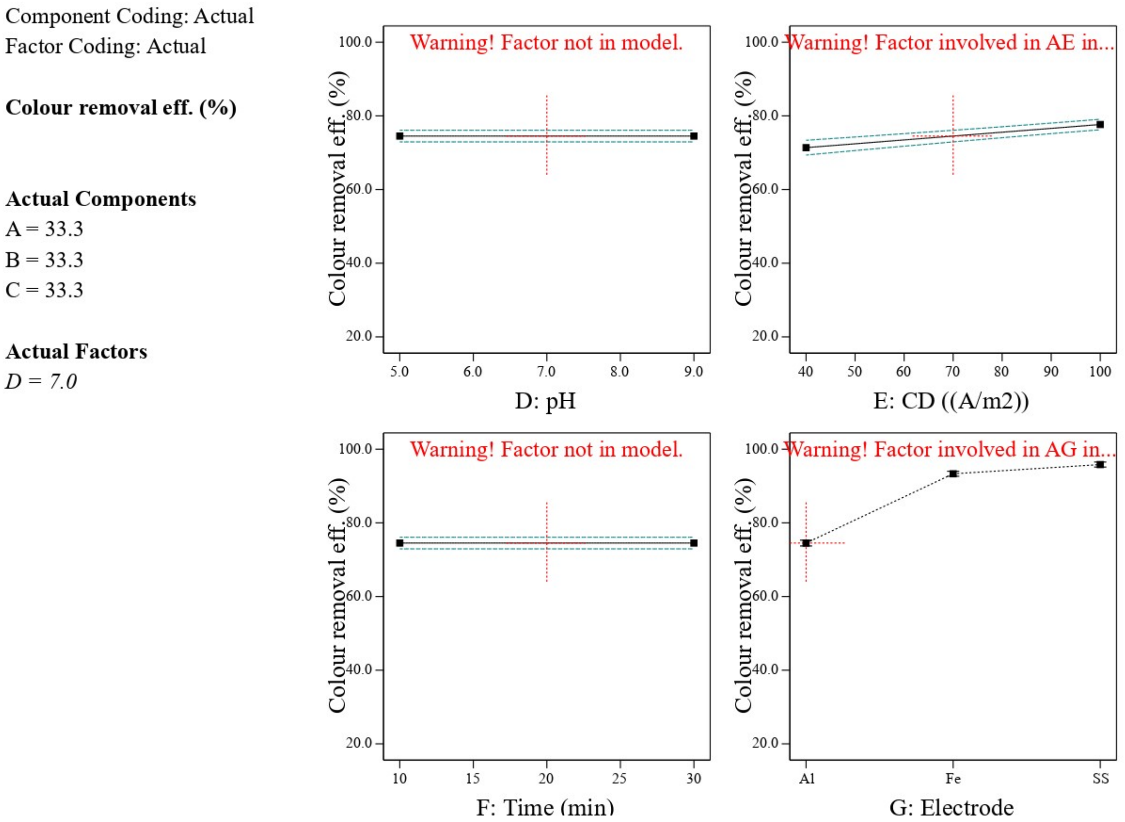

Fig. S4 One factor plots showing the effect of pH, current density, treatment time and electrode type on colour removal of mixture of dyes (33.3 mg/L each)

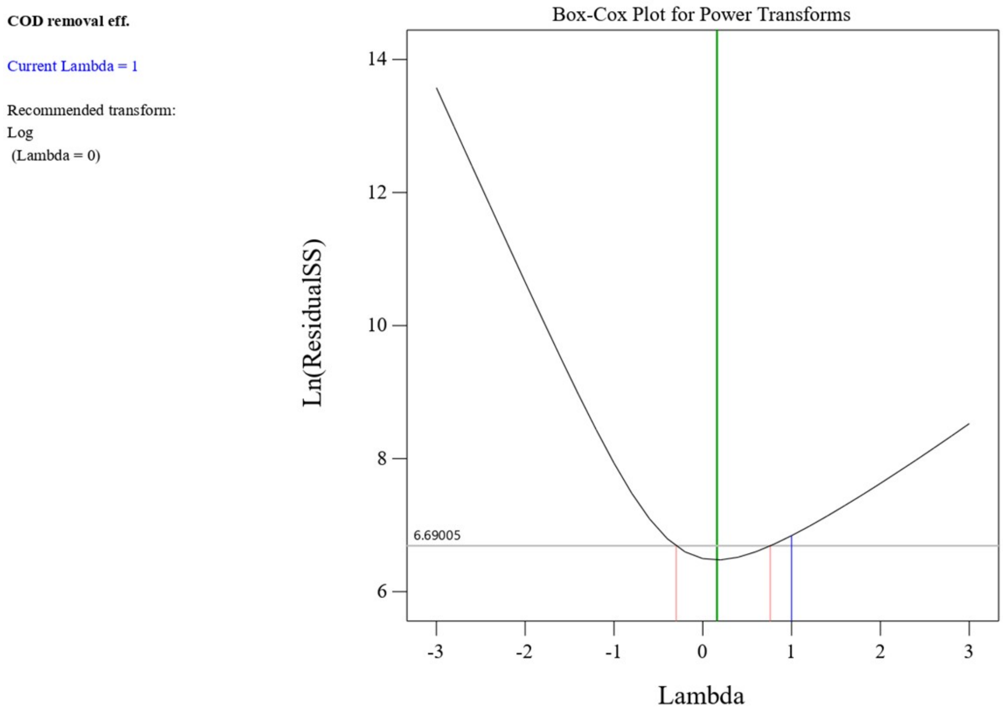

Fig. S5 Box-Cox plot for power transform suggesting natural log transformation

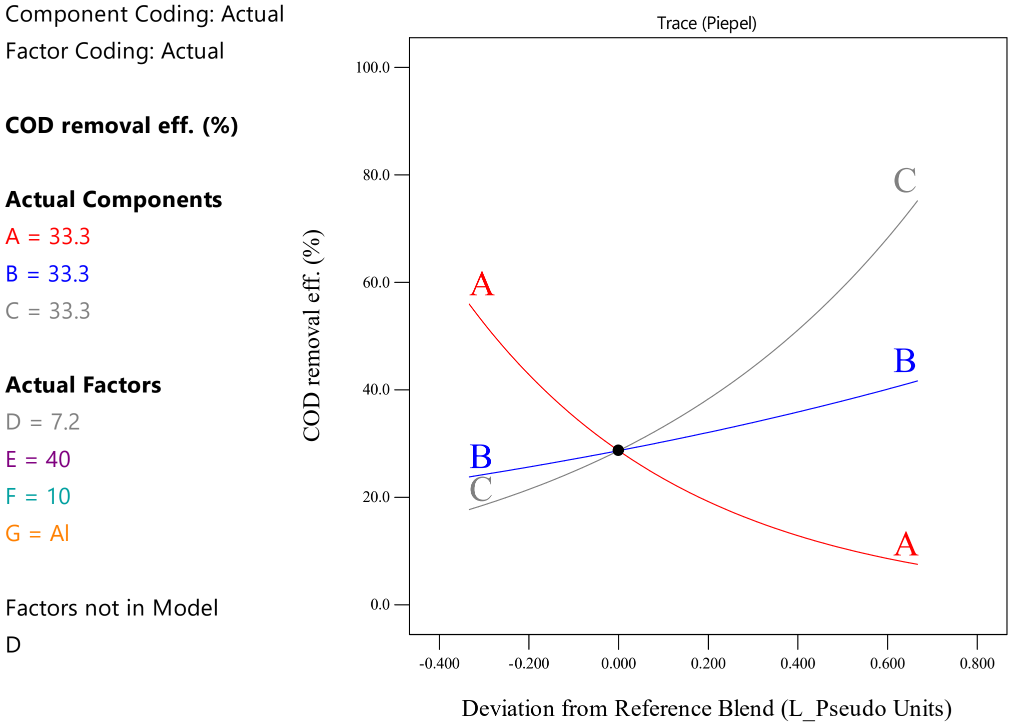

Fig. S6a Trace plot showing the effect of dyes concentrations using aluminium on COD removal efficiency of dye mixtures *(A, B, C= Dye concentration, D=pH, E=Current density, F=Treatment time, G=Electrode pair)*

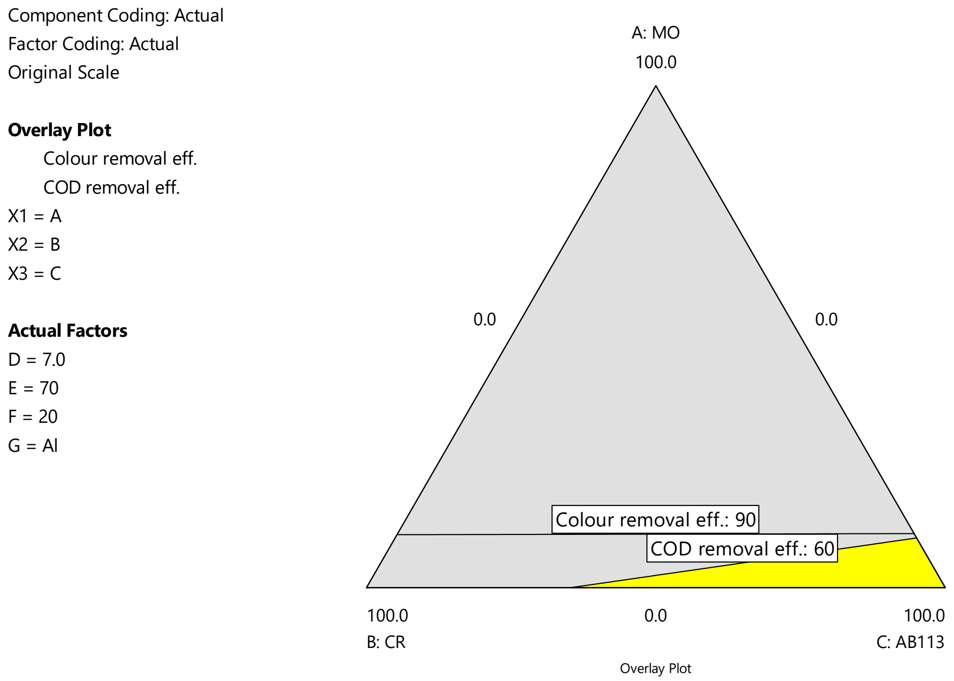

Fig. S6b Overlay plot showing the effect of dyes concentrations using aluminium electrodes on COD removal efficiency of dye *(A, B, C= Dye concentration, D=pH, E=Current density, F=Treatment time, G=Electrode pair)*

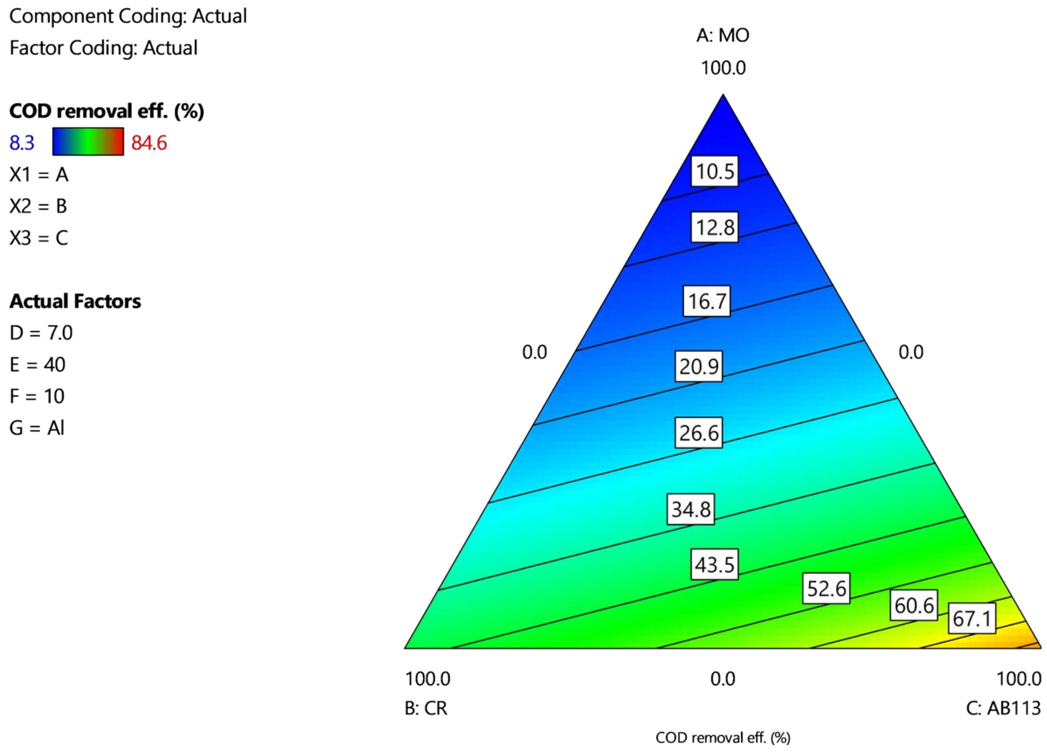

Fig. S6c Two-dimensional contour plot showing the effect of dyes concentrations using aluminium on COD removal efficiency of dye mixtures *(A, B, C= Dye concentration, D=pH, E=Current density, F=Treatment time, G=Electrode pair)*

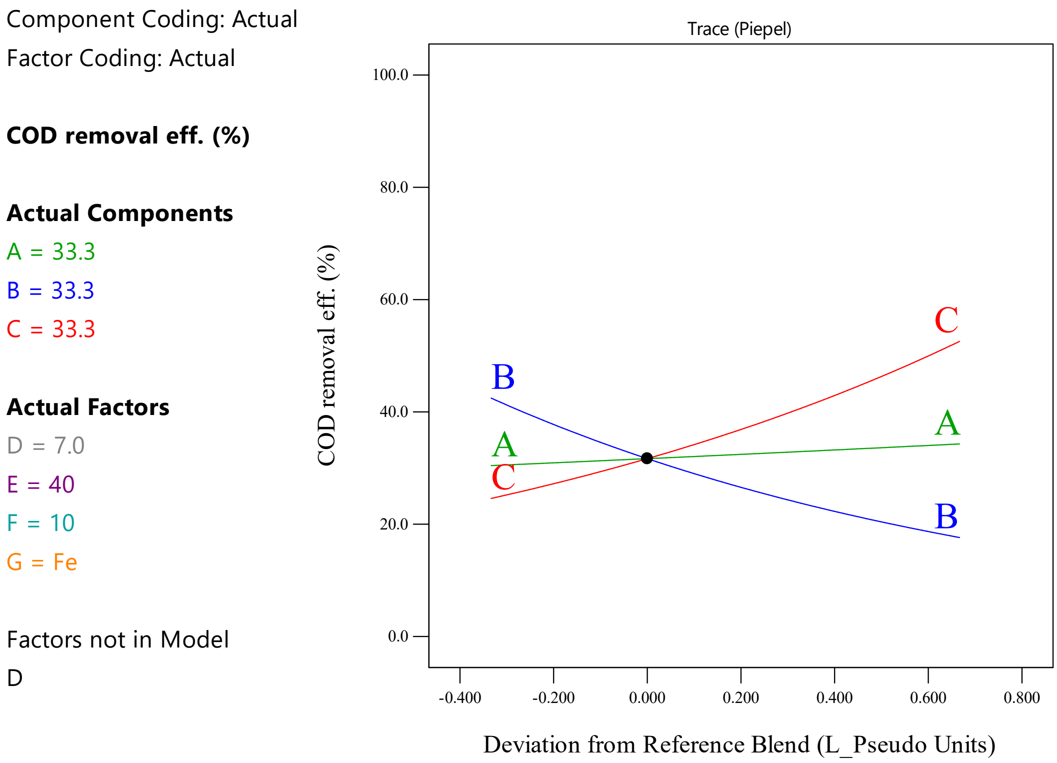

Fig. S7a Trace plot showing the effect of dyes concentrations using iron electrodes on COD removal efficiency of dye mixtures *(A, B, C= Dye concentration, D=pH, E=Current density, F=Treatment time, G=Electrode pair)*

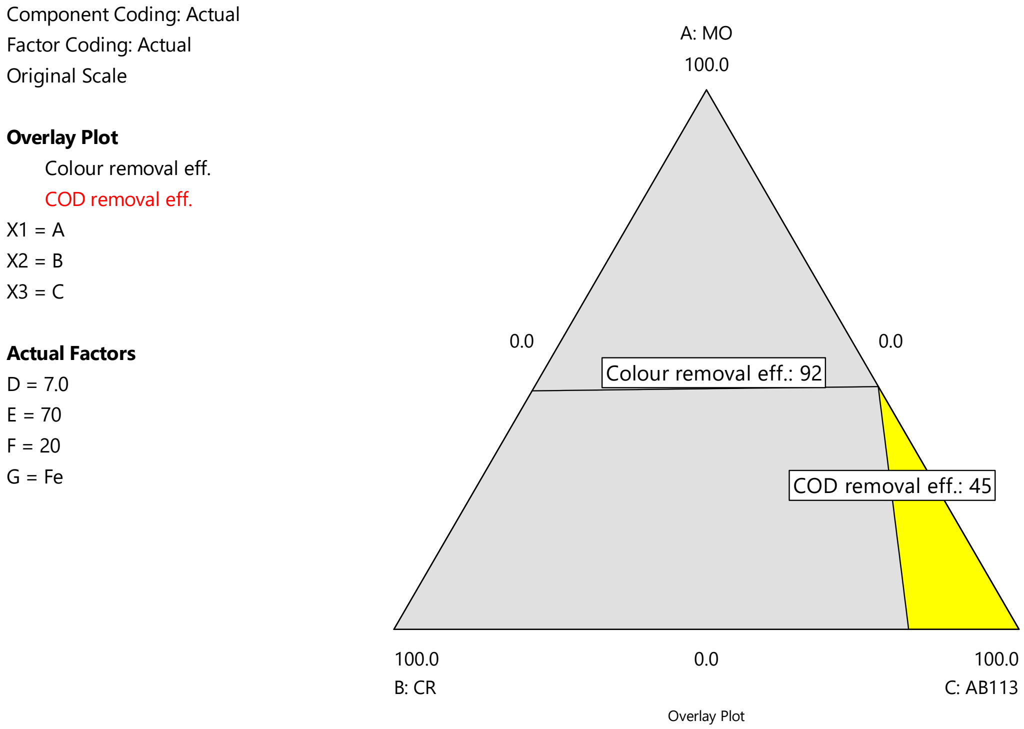

Fig. S7b Overlay plot showing the effect of dyes concentrations using iron electrodes on COD removal efficiency of dye mixtures *(A, B, C= Dye concentration, D=pH, E=Current density, F=Treatment time, G=Electrode pair)*

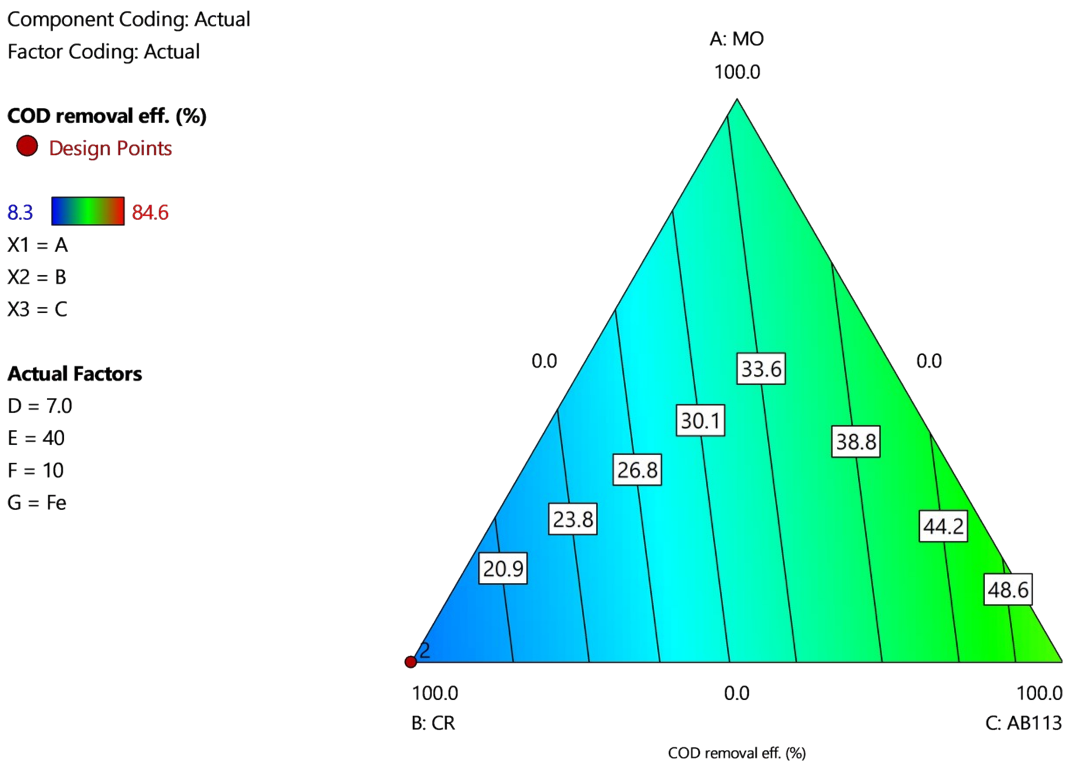

Fig. S7c 2-D contour plot showing the effect of dyes concentrations using iron on COD removal efficiency of dye mixtures *(A, B, C= Dye concentration, D=pH, E=Current density, F=Treatment time, G=Electrode pair)*

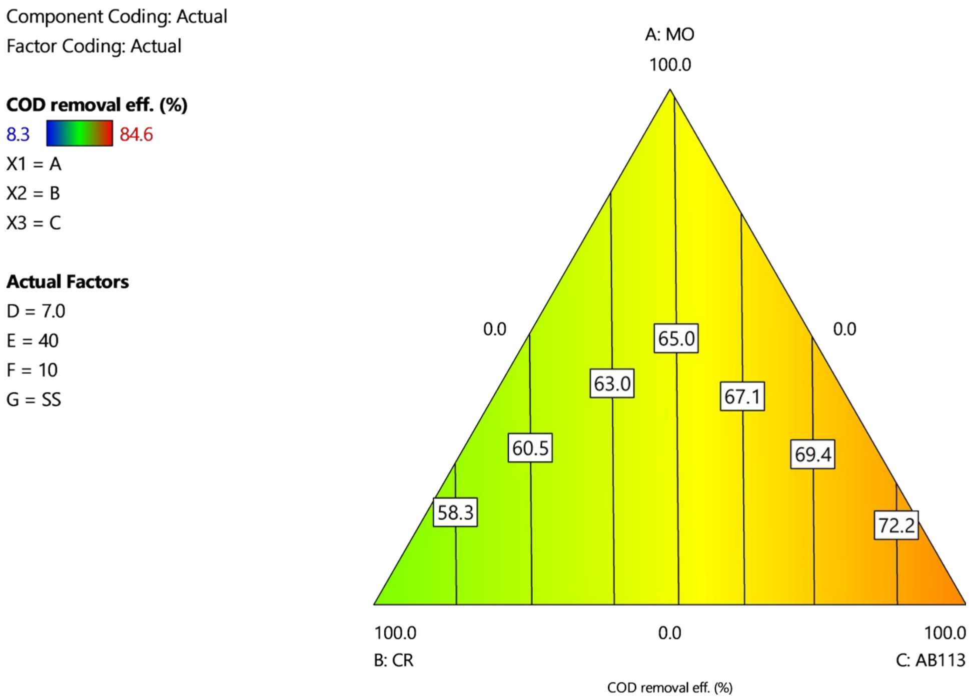

Fig. S8 Two-dimensional contour plot showing the effect of dyes concentrations using stainless steel on COD removal efficiency of dye mixtures *(A, B, C= Dye concentration, D=pH, E=Current density, F=Treatment time, G=Electrode pair)*

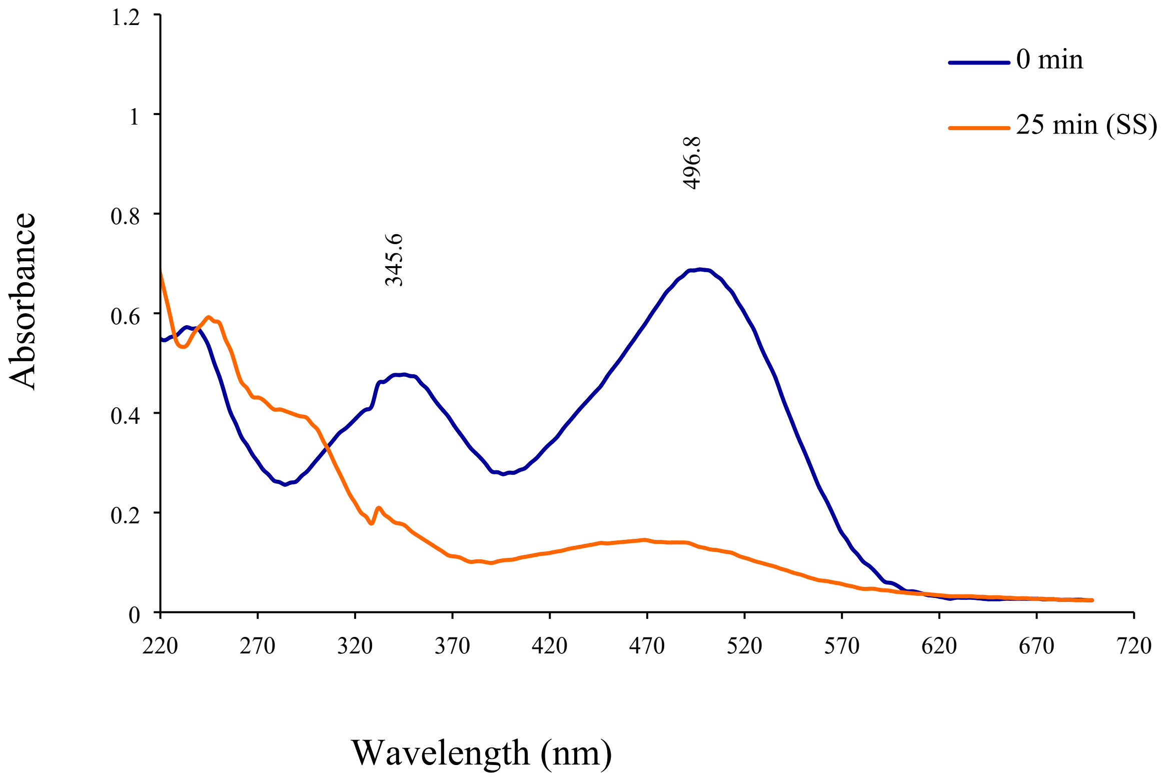

Fig. S9 Absorption spectra of mixtures of dyes [MO: 2 mg/L, CR: 98 mg/L, AB113: 0 mg/L] at start (0 min) and 25 min treatment time. Other conditions are pH= 5, current density= 78 A/m^2 with SS electrode pair

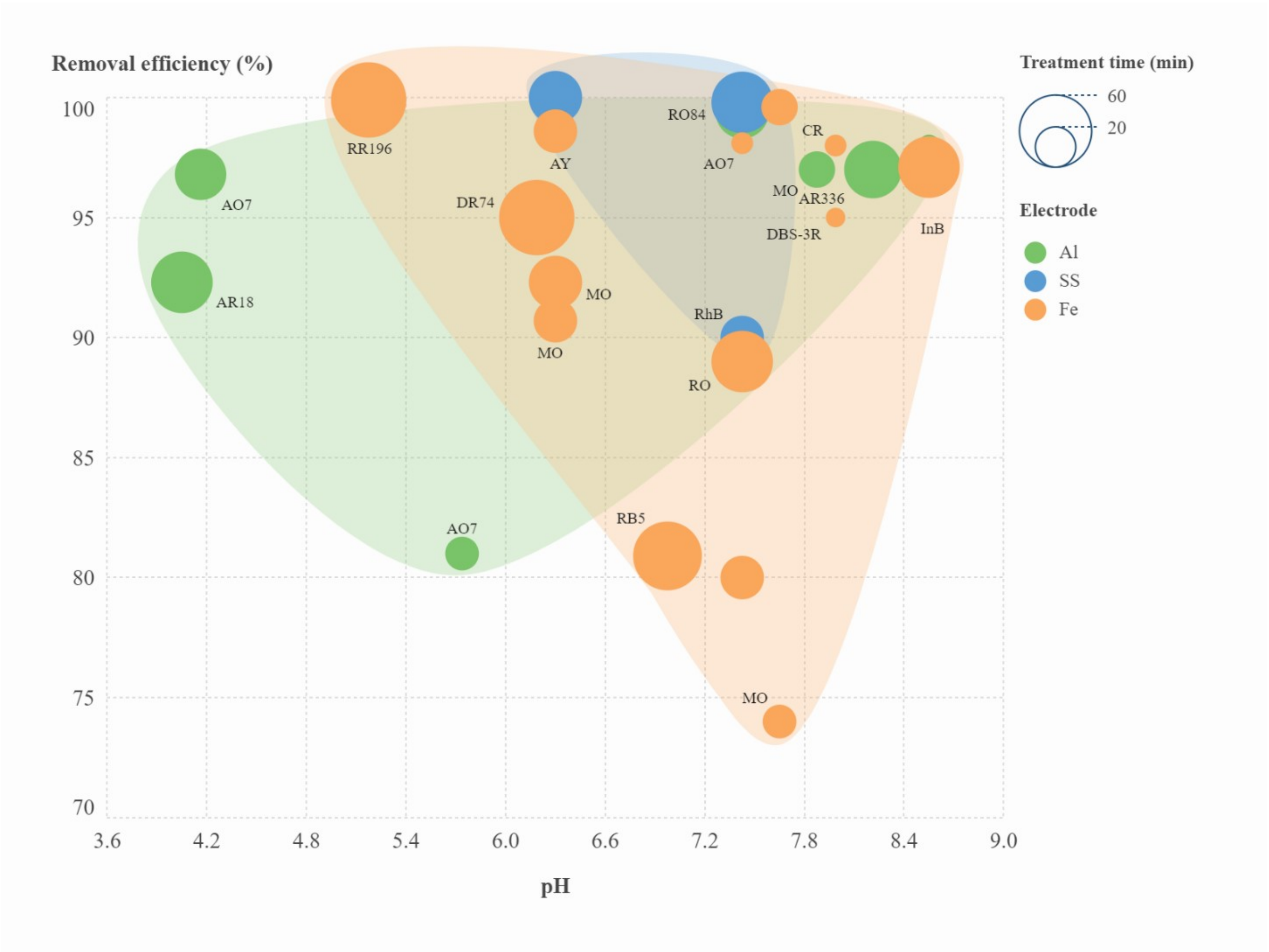

Fig. S10 Convex hull graph showing effect of pH on colour removal efficiency with size of the circle represents treatment time and colour of the circle represent the electrode type along with dye name along the circle (details in table S4)

Table S1 ANOVA table and model statistics for colour removal efficiency (a) with outliers (b) after removing outliers

(a) With outliers

Source	SS	D_f	MS	F-value	p-value Prob>F
Model	9853.6	6	1642.2	91.1	< 0.0001*
Linear mixture	2708.6	2	1354.3	75.1	< 0.0001*
$X_1 \times X_5$	257.9	1	257.9	14.3	0.0011*
$X_1 \times X_6$	157.0	1	157.0	8.7	0.0076*
$X_1 \times X_7$	5474.1	2	2737.0	151.9	< 0.0001*
Lack of Fit	373.1	16	23.3	22.7	0.0014*

Model Statistics

Standard deviation = 4.2, Mean = 89.0, C.V. % = 4.7, PRESS = 2016.4

R^2 = 0.963, Adjusted R^2 = 0.952, Predicted R^2 = 0.802, Adequate Precision = 34.317

(b) After removing outliers

Source	Sum of Square	Degree of freedom	Mean Sum of Square	F-value	p-value Prob>F
Model	3813.5	5	762.7	206.0	< 0.0001*
Linear mixture	785.7	2	392.8	106.1	< 0.0001*
X_1 x X_5	298.7	1	298.7	80.7	< 0.0001*
X_1 x X_7	3027.3	2	1513.6	408.8	< 0.0001*
Lack of Fit	68.9	15	4.5	4.4	0.0532#

Model Statistics

Standard deviation = 1.92, Mean = 93.2, C.V. % = 2.0, PRESS = 248.1

R^2 = 0.981, Adjusted R^2 = 0.976, Predicted R^2 = 0.936, Adequate Precision = 69.15

X_1= Methyl orange, X_2= Congo red, X_3= Acid blue 113, X_5= Current density, X_6= Treatment time, X_7= Electrode

* significant at $p \leq 0.05$, # not-significant at $p \leq 0.05$

Table S2 ANOVA table and model statistics for COD removal efficiency before transformation

Source	Sum of Square	Degree of freedom	Mean Sum of Square	F-value	p-value Prob>F
Model	15063.08	9	1673.68	32.15	< 0.0001*
Linear Mixture	2859.03	2	1429.52	27.46	< 0.0001*
$X_1 \times X_7$	6561.34	2	3280.67	63.01	< 0.0001*
$X_2 \times X_6$	182.19	1	182.19	3.50	0.0777#
$X_2 \times X_7$	4436.10	2	2218.05	42.60	< 0.0001*
$X_3 \times X_7$	791.24	2	395.62	7.60	0.0041*
Lack of fit	875.71	13	67.36	5.48	0.0357*

Model Statistics

Standard deviation = 7.2, Mean = 51.2, C.V. % = 14.0, PRESS = 2154.3

R^2 = 0.941, Adjusted R^2 = 0.912, Predicted R^2 = 0.865, Adequate Precision = 17.26

X_1= Methyl orange, X_2= Congo red, X_3= Acid blue 113, X_5= Current density, X_6= Treatment time, X_7= Electrode

* significant at $p \leq 0.05$, # not-significant at $p \leq 0.05$

Table S3 Comparison of present study with previous studies related to electrocoagulation removal of dyes mixture

	Dyes	Electrode pair	Initial dye conc. (mg/L)	Optimized parameters			Colour removal efficiency (%)	Reference
				pH	Current density (A/m^2)	Treatment time (min)		
1	• Acid green 20<br>• Reactive yellow 17	Al	-	2.1	1000	60	86.5<br>92.2	[25]
2	• Yellow terasil 4G<br>• Red terasil 343<br>• Blue terasil 3R02<br>• Red S3B<br>• Yellow SPD<br>• Blue BRFS	Fe	400	7.5	400	45	80.0	[26]
3	• Acid blue 113<br>• Acid blue 29<br>• Brilliant green	SS	450*	7.0	220	35	55.0<br>67.5 (COD)	[27]
4	• Reactive red<br>• Reactive blue 221<br>• Reactive yellow 145	SS	303*	11.5	220	30	100<br>63.0 (COD)	[28]
5	• Disperse blue 56<br>• Basic yellow 28	Fe	100	7.0	108	10	85.0	[29]
**6**	• **Methyl orange**<br>• **Congo red**<br>• **Acid blue 113**	**SS**	**100**	**5.3**	**100**	**30**	**99.0**<br>**81.9 (COD)**	**Own study**
7	• Reactive yellow 145<br>• Acid violet 92	Fe	100	4	55.6	10	82% (COD)	[30]

*initial COD concentration

Table S4 Literature review of optimized process parameters for electrocoagulation removal of dyes along with colour removal efficiency using different electrode pairs

	Electrode type	Dye	Dye (symbol)	Conc. (mg/L)	pH	CD (A/m^2)	Time (min)	Colour removal efficiency (%)	Reference	Reference number
1	Al	Methylene blue	MeB	30	7.0	50	30	99.4	Liu (2019)	[31]
2	Al	Disperse blue 56	DB56	25	8.0	200	5	98.0	Dassa (2020)	[32]
3	Al	Acid orange 7	AO7	150	5.5	229	12	81.0	Taheri (2022)	[37]
4	Al	Acid orange 7	AO7	125	4.1	239	28	96.8	Taheri (2015)	[34]
5	Al	Acid blue 113	AB113	100	7.0	250	5	99.5	Taheri (2014)	[33]
6	Al	Acid red 18	AR18	100	4.0	290	40	92.3	Khosravi (2016)	[36]
7	Al	Acid red 336	AR336	300	7.7	300	35	97.0	Amour (2016)	[35]
8	Al	Methyl orange	MO	125	7.4	1850*	14	97.0	Pi (2014)	[20]
9	Al	Congo red	CR	65	9.5*	2040*	25	89.3	Sankar (2020)	[38]
10	SS	Brilliant green	BG	338	6.0	60	30	100	Marquez (2022)	[24]
11	SS	Rhodamine B	RhB	50	7.0	72	20	90.0	Adeogun (2017)	[23]
12	SS	Reactive orange 84	RO84	300	7.0	110	40	99.8	Yuksel (2013)	[21]
13	Fe	Methyl orange	MO	25	8.0	30	40	97.1	Abbas (2022)	[5]
14	Fe	Indigo blue	InB	50	8.1	56	120*	95.0	De Maman (2022)	[43]
15	Fe	Reactive red 196	RR196	50	5.0	62	60	99.9	Assadi (2016)	[41]
16	Fe	Methyl orange	MO	15	7.2	64	12	74.0	Irki (2017)	[42]
17	Fe	Methylene blue	MB	50	7.0	80	20	80.0	Mahmoud (2013)	[40]
18	Fe	Reactive red 24	RR24	100	7.2	100	14	99.6	Ghalwa (2016)	[45]
19	Fe	Methyl orange	MO	134	6.0	100	30	92.3	Wu (2021)	[48]
20	Fe	Disperse red 74	DR74	40	5.9	123	60	95.0	Houssini (2020)	[47]
21	Fe	Reactive orange	RO	300	7.0	130	40	89.0	Yuksel (2013)	[21]
22	Fe	Congo red	CR	50	7.5	150	5	98.0	Mohammadlou (2014)	[44]
23	Fe	Reactive Black 5	RB5	40	6.6	250	50	80.9	Mook (2017)	[46]
24	Fe	Disperse brown S-3R	DBS-3R	250	7.5	300	4	95.0	Bektas *et al.*, 2019	[49]
25	Fe	Alizarin yellow	AY	100	6.0	300	20	98.6	Liu *et al.*,2022	[50]
26	Fe	Methyl orange	MO	100	6.0	300	20	90.7	Liu *et al.*,2022	[50]
27	Fe	Acid orange 7	AO7	50	7.0	350	5	98.1	Chafi *et al.*, 2011	[51]

** values are not used in Fig. 5 and Fig. S10 for better visualization of data.*
